# Supplementary material for: The impact of anorexia nervosa and BMI polygenic risk on childhood growth: A 20-year longitudinal population-based study
Source: Am J Hum Genet. 2022 Jun 14;109(7):1242–54. doi: 10.1016/j.ajhg.2022.05.005 (PMC9300758; doi:10.1016/j.ajhg.2022.05.005)
Supplement: Document S1. Supplemental materials and methods, Figures S1 and S2, and Tables S1–S6 [file mmc1.pdf]

The American Journal of Human Genetics, Volume *109*

Supplemental information

The impact of anorexia nervosa and BMI polygenic risk on childhood growth: A 20-year longitudinal population-based study

Mohamed Abdulkadir, Christopher Hübel, Moritz Herle, Ruth J.F. Loos, Gerome Breen, Cynthia M. Bulik, and Nadia Micali

Supplemental information

Supplemental figures

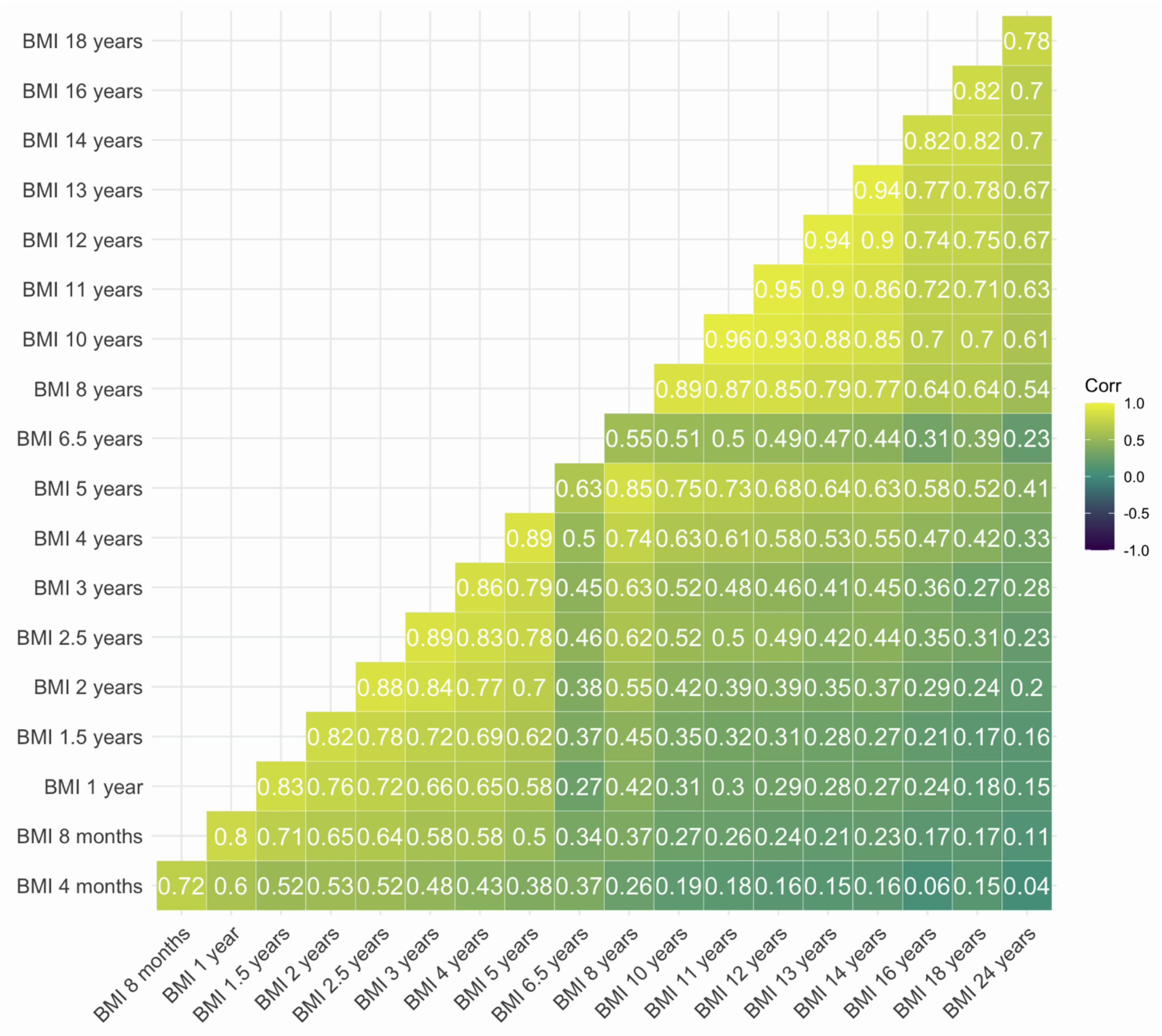

Figure S1: Correlation matrix of body mass index (BMI; weight in kilograms/height^2 in meters) across childhood and adolescence in the Avon Longitudinal Study of Parents and Children (ALSPAC). Numerous measurements on weight and height were collected from different sources (i.e., routine clinic visits, information collected from midwives, linkage to child health records) between birth and age 24 years. Information on weight was collected at research clinic visits annually up to the age 14 years and further clinic measurements at the ages 16, 18, and 24 years using the Tanita Body Fat Analyzer (Tanita TBFUK Ltd.) to the nearest 50 grams. During the same clinic visits, height (standing) was measured to the nearest millimeter with shoes and socks removed using a Holtain stadiometer (Holtain Ltd, Crymych, Pembs, UK). Information on child and adolescent BMI (weight in kilograms / height squared in meters) was derived using weight and height measurement obtained during clinic visits.

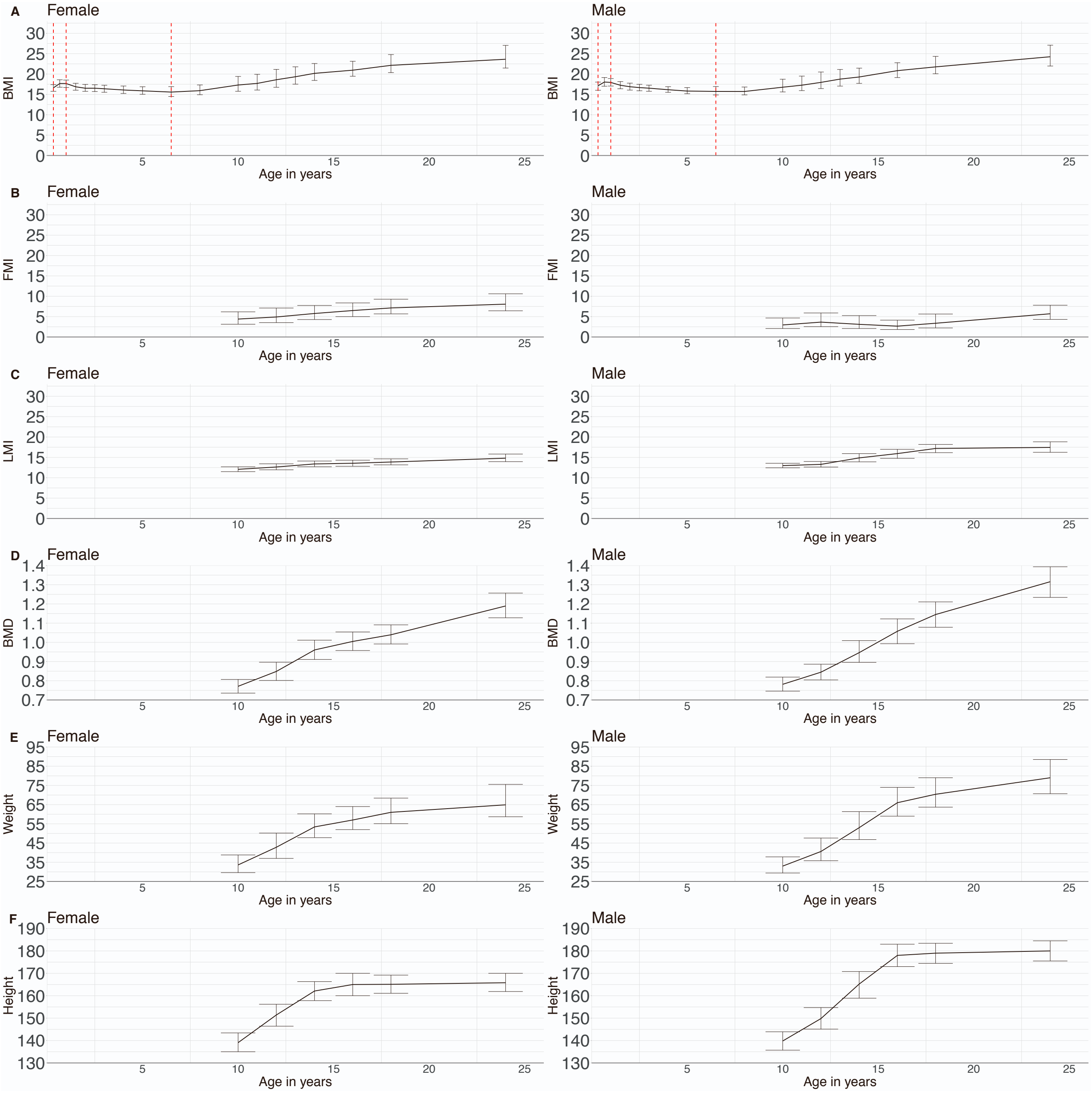

Figure S2: A. Median of body mass index (BMI; weight in kilograms/height^2 in meters) trajectories across childhood and adolescence. The BMI trajectory was derived using spline modeling. Prior to deriving the trajectory. For the spline modeling three spline points (knots shown in red) were placed: knot 1, between age 4 months and 1 year; knot 2, between age 1 year and 6.5 years; and knot 3, between age 6.5 year and 24 years. Intercept reflect BMI value at age 4 months. **B-F** Median fat mass index (FMI; fat mass in kilograms/height^2 in meters), lean mass index (LMI; lean mass in kilograms/height^2 in meters, bone mineral density (BMD; in gram/cm^2), weigh, and height trajectories across childhood and adolescents, respectively. FMI, LMI, BMD, weight, and height trajectory parameters were derived using mixed effects models [7]. Error bars represent the interquartile range.

Supplemental tables

Table S1: Descriptive of the body composition measures of the Avon Longitudinal Study of Parents and Children (ALSPAC)[a]

		Female		Male	
**Body composition measure**	**Age (months)**	**N**	**Median (IQR)**	**N**	**Median (IQR)**
**BMI** [b]	4	337	16.61 (15.76, 17.41)	363	17.12 (16.02, 18.02)
	8	438	17.67 (16.76, 18.6)	491	18.04 (17.01, 19.14)
	12	423	17.67 (16.69, 18.52)	467	17.94 (17.05, 18.89)
	18	405	16.87 (16.03, 17.73)	440	17.25 (16.38, 18.15)
	24	370	16.52 (15.75, 17.47)	411	16.89 (16.06, 17.77)
	30	384	16.53 (15.70, 17.37)	422	16.67 (15.89, 17.48)
	36	385	16.38 (15.53, 17.25)	417	16.53 (15.77, 17.26)
	48	372	16.08 (15.23, 17.06)	419	16.15 (15.52, 16.9)
	60	367	15.88 (15.03, 16.87)	403	15.82 (15.17, 16.67)
	78	544	15.57 (14.49, 16.91)	534	15.73 (14.86, 16.93)
	96	3,130	15.92 (14.92, 17.32)	3,224	15.72 (14.88, 16.82)
	120	3,072	17.31 (15.77, 19.41)	3,026	16.77 (15.61, 18.73)
	132	2,977	17.7 (16.07, 19.94)	2,923	17.26 (15.93, 19.50)
	144	2,902	18.6 (16.74, 21.14)	2,797	17.96 (16.43, 20.50)
	156	2,742	19.34 (17.52, 21.78)	2,630	18.76 (17.04, 21.12)
	168	2,433	20.17 (18.43, 22.58)	2,394	19.28 (17.71, 21.45)
	192	1,496	20.95 (19.47, 23.14)	1,289	20.83 (19.14, 22.78)
	216	1,986	22.14 (20.35, 24.78)	1,681	21.76 (20.08, 24.34)
	288	1,671	23.63 (21.47, 27.04)	1,131	24.25 (21.97, 27.09)
**FMI** [c]	120	2,932	4.40 (3.14, 6.18)	2,880	2.96 (2.08, 4.67)
	144	2,862	4.91 (3.52, 7.11)	2,750	3.65 (2.53, 5.90)
	168	2,406	5.77 (4.28, 7.74)	2,353	3.11 (2.09, 5.26)
	192	1,266	6.50 (5.00, 8.36)	1,066	2.67 (1.83, 4.14)
	216	1,895	7.13 (5.68, 9.28)	1,624	3.37 (2.22, 5.64)
	288	1,618	8.07 (6.43, 10.61)	1,100	5.70 (4.32, 7.80)
**LMI** [c]	120	2,932	12.07 (11.5, 12.69)	2,880	12.98 (12.43, 13.55)
	144	2,862	12.64 (11.95, 13.42)	2,750	13.28 (12.63, 14.00)
	168	2,406	13.39 (12.70, 14.10)	2,353	14.87 (13.90, 15.92)
	192	1,266	13.56 (12.81, 14.3)	1,066	15.93 (14.76, 16.98)
	216	1,895	13.86 (13.17, 14.64)	1,624	17.19 (16.16, 18.18)
	288	1,618	14.8 (13.95, 15.82)	1,100	17.45 (16.25, 18.83)

**BMD** [c]	120	2,965	0.77 (0.74, 0.81)	2,900	0.78 (0.75, 0.82)
	144	2,865	0.85 (0.80, 0.90)	2,756	0.84 (0.80, 0.89)
	168	2,406	0.96 (0.91, 1.01)	2,354	0.95 (0.90, 1.01)
	192	2,050	1.00 (0.96, 1.05)	1,952	1.06 (0.99, 1.12)
	216	1,902	1.04 (0.99, 1.09)	1,634	1.14 (1.08, 1.21)
	288	1,619	1.19 (1.13, 1.26)	1,101	1.32 (1.23, 1.39)
**Weight (in kilograms)** [d]	120	3,105	33.60 (29.6, 38.8)	3,045	33 (29.40, 37.80)
	144	2,904	42.80 (37.00, 50.20)	2,801	40.60 (35.80, 47.60)
	168	2,433	53.40 (47.80, 60.20)	2,394	53.00 (46.80, 61.35)
	192	1,599	57.00 (52.00, 64.00)	1,330	66.00 (59.00, 74.00)
	216	1,986	61.00 (55.10, 68.40)	1,683	70.40 (63.70, 79.00)
	288	1,671	64.90 (58.70, 75.55)	1,131	79.00 (70.70, 88.50)
**Height (in centimeters)** [d]	120	3,074	139.05 (135.0, 143.4)	3,027	139.8 (135.7, 143.9)
	144	2,904	151.4 (146.4, 156.2)	2,797	149.8 (145.1, 154.7)
	168	2,438	162.1 (157.8, 166.3)	2,394	165.2 (158.9, 170.8)
	192	1,666	165.0 (160.0, 170.0)	1,361	178.0 (173.0, 183.0)
	216	1,988	165.1 (161.1, 169.2)	1,683	179.0 (174.5, 183.4)
	288	1,672	165.8 (161.9, 170.0)	1,131	180.0 (175.5, 184.5)

IQR = interquartile range; BMI, body mass index (weight in kilograms/height^2 in meters); FMI, fat mass index (fat mass in kilograms/height^2 in meters); LMI, lean mass index (lean mass in kilograms/height^2 in meters); BMD, bone mineral density.

[a]Full description of the Avon Longitudinal Study of Parents and Children (ALSPAC) is described elsewhere; [2–6].

[b]BMI was calculated using objectively measured weight and height that were collected from different sources (i.e., routine clinic visits, information collected from midwives, linkage to child health records) between birth and age 24 years. Information on weight was collected at research clinic visit annually up to the age 14 years and further clinic measurements at the ages 16, 18, and 24 years using the Tanita Body Fat Analyzer (Tanita TBFUK Ltd.) to the nearest 50 grams. During the same clinic visits, height (standing) was measured to the nearest millimeter with shoes and socks removed using a Holtain stadiometer (Holtain Ltd, Crymych, Pembs, UK).

[c]Fat mass, lean mass, and bone mineral density (BMD) were derived using a Lunar Prodigy dual emission x ray absorptiometry (DEXA) scanner (GE Medical Systems Lunar, Madison, WI, USA). Fat mass index (FMI) and lean mass index (LMI) were calculated by dividing each measure (in kilograms) by height^2 (in meters). The bone mineral density (BMD) is calculated for the whole body excluding the head values.

[d]For uniformity of the analyses, we restricted analyses of weight and height to measurements taken at the same ages FMI, LMI, and BMD were measured.

Table S2: Sex comparisons of the associations of the anorexia nervosa polygenic score with body composition in the Avon Longitudinal Study of Parents and Children (ALSPAC) [a]

Trajectory	Parameter	Beta female [b]	Beta on the log scale female [b]	Standard error female	Beta male [b]	Beta Male on the log scale [b]	Standard error male	% difference in slope females compared to males	Z	P
BMI	Slope age 4 months - 1 year	1.0000	7.48E-07	2.42E-06	1.0000	1.01E-06	2.20E-06	-35.03	-0.08	0.94
BMI	Slope age 1 year - 6.5 years	1.0000	1.85E-05	1.68E-05	1.0000	-5.87E-06	1.62E-05	131.73	1.04	0.29
BMI	Slope age 6.5 years - 24 years	1.0000	-4.00E-05	1.22E-05	1.0000	-2.31E-05	1.08E-05	42.25	-1.03	0.30
FMI	Slope age 10 years - 24 years	0.9919	-8.09E-03	7.55E-03	0.9942	-5.79E-03	9.54E-03	28.43	-0.19	0.85
LMI	Slope age 10 years - 24 years	0.9973)	-2.69E-03	1.49E-03	0.9993	-6.51E-04	1.68E-03	75.80	-0.91	0.36
BMD	Slope age 10 years - 24 years	0.9960	-4.02E-03	1.59E-03	0.9986	-1.44E-03	1.84E-03	64.18	-1.06	0.29
Weight	Slope age 10 years - 24 years	0.9937	-6.30E-03	3.62E-03	0.9956	-4.38E-03	4.05E-03	30.48	-0.35	0.73
Height	Slope age 10 years - 24 years	0.9990)	-1.02E-03	8.25E-04	0.9991	-9.10E-04	1.07E-03	10.78	-0.08	0.94

[a] Full description of the Avon Longitudinal Study of Parents and Children is described elsewhere; [2–6].

[b] Comparison of the betas were done on the log scale as the outcomes were log transformed. Reported here are the non-log transformed and the log transformed betas.

Table S3: Post-hoc power analysis of the anorexia nervosa and body mass index polygenic score.

	AN PGS			BMI PGS	
Trajectory	N	Cov12	Power	Cov12	Power
BMI	3072	-0.32	1	0.95	1
FMI	2932	-0.33	1	0.90	1
LMI	2932	-0.12	1	0.54	1
BMD	2965	0.06	0.97	0.39	1
Weight	3105	-0.23	1	0.82	1
Height	3074	-0.11	1	-0.17	1

AN = anorexia nervosa; PGS = polygenic score ; BMI = body mass index; N = sample size target sample (Avon Longitudinal Study of Parents and Children); Cov12 = Covariance between genetic effect sizes in the two samples; Power = Power of the Chi-squared test of association between polygenic score and target trait.

Table S4: Sex comparisons of the associations of the body mass index polygenic score with body composition in the Avon Longitudinal Study of Parents and Children (ALSPAC) [a]

Trajectory	Parameter	Beta female [b]	Beta on the log scale female [b]	Standard error female	Beta male [b]	Beta on the log scale male [b]	Standard error male	% difference in slope females compared to males	Z	P
BMI	Slope age 4 months - 1 year	1.0000	3.92E-05	2.28E-06	1.0000	2.99E-05	2.09E-06	23.72	2.99	0.003
BMI	Slope age 1 year - 6.5 years	1.0002	1.83E-04	1.68E-05	1.0002	1.93E-04	1.60E-05	-5.46	-0.45	0.65
BMI	Slope age 6.5 years - 24 years	1.0002	1.80E-04	1.22E-05	1.0002	1.57E-04	1.07E-05	12.78	1.42	0.15
FMI	Slope age 10 years - 24 years	1.1673	1.55E-01	6.90E-03	1.2040	1.86E-01	8.69E-03	-20.00	-2.79	0.005
LMI	Slope age 10 years - 24 years	1.0211	2.09E-02	1.40E-03	1.0185	1.83E-02	1.62E-03	12.44	1.19	0.23
BMD	Slope age 10 years - 24 years	1.0164	1.63E-02	1.54E-03	1.0142	1.41E-02	1.78E-03	13.50	0.9	0.37
Weight	Slope age 10 years - 24 years	1.0642	6.22E-02	3.37E-03	1.0584	5.68E-02	3.79E-03	8.68	1.08	0.28
Height	Slope age 10 years - 24 years	1.0016	1.62E-03	8.35E-04	1.0023	2.35E-03	1.06E-03	-45.06	-0.53	0.59

[a] Full description of the Avon Longitudinal Study of Parents and Children is described elsewhere; [2–6].

[b] Comparison of the betas were done on the log scale as the outcomes were log transformed. Reported here are the non-log transformed and the log transformed betas.

Table S5: Sample size of the combined anorexia nervosa and body mass index polygenic scores groups in the Avon Longitudinal Study of Parents and Children[a]

	**Female**	**Male**
**PGS category** [b]	**N**	**N**
Low AN / high BMI	778	702
High AN / high BMI	137	114
High AN / low BMI	765	715
Low AN / low BMI	2,749	2,694

[a] Full description of the Avon Longitudinal Study of Parents and Children is described elsewhere; [2–6].

[b] Categorical variable derived from dichotomizing the AN-PGS and the BMI-PGS. For both the AN and the BMI PGS, individuals with scores at or greater than the 8th decile point were regarded as the "high PGS group" while those with scores lower were considered "low PGS group". From the dichotomized AN-PGS and BMI-PGS we were able to create a categorical variable with four levels (1) low AN-PGS / low BMI-PGS (2) high AN-PGS / low BMI-PGS (3) high AN-PGS / high BMI-PGS (4) low AN-PGS / high BMI-PGS. The "low AN-PGS/low BMI-PGS" group was used as the reference category in the extreme analyses.

Table S6: Associations of the combined anorexia nervosa and body mass index polygenic score with the body composition measures stratified for biological sex using linear mixed models in the Avon Longitudinal Study of Parents and Children: Post-hoc analyses of the BMI trajectory [a b]

		**Female**				**Male**			
**Trajectory**	**Comparison** [b]	**Beta**	**SE**	**Z ratio**	**P** [c]	**Beta**	**SE**	**Z ratio**	**P** [c]
**BMI**	Low AN PGS and low BMI PGS - High AN PGS and low BMI PGS	0.002	0.006	0.270	0.99	-0.001	0.006	-0.153	1
	Low AN PGS and low BMI PGS - High AN PGS and high BMI PGS	-0.102	0.014	-7.044	**<0.001***	-0.098	0.013	-7.775	**<0.001***
	Low AN PGS and low BMI PGS - Low AN PGS and high BMI PGS	-0.093	0.006	-14.375	**<0.001***	-0.071	0.006	-12.308	**<0.001***
	High AN PGS and low BMI PGS - High AN PGS and high BMI PGS	-0.104	0.015	-6.807	**<0.001***	-0.097	0.013	-7.315	**<0.001***
	High AN PGS and low BMI PGS - Low AN PGS and high BMI PGS	-0.095	0.008	-11.690	**<0.001***	-0.071	0.007	-9.708	**<0.001***
	High AN PGS and high BMI PGS - Low AN PGS and high BMI PGS	0.009	0.015	0.567	0.94	0.026	0.013	1.990	0.19
**FMI**	Low AN PGS and low BMI PGS - High AN PGS and low BMI PGS	0.013	0.018	0.720	0.89	-0.015	0.025	-0.613	0.93
	Low AN PGS and low BMI PGS - High AN PGS and high BMI PGS	-0.274	0.042	-6.583	**<0.001***	-0.390	0.053	-7.304	**<0.001***
	Low AN PGS and low BMI PGS - Low AN PGS and high BMI PGS	-0.245	0.019	-13.125	**<0.001***	-0.323	0.025	-13.135	**<0.001***
	High AN PGS and low BMI PGS - High AN PGS and high BMI PGS	-0.287	0.044	-6.563	**<0.001***	-0.375	0.056	-6.675	**<0.001***
	High AN PGS and low BMI PGS - Low AN PGS and high BMI PGS	-0.258	0.023	-11.100	**<0.001***	-0.308	0.031	-10.016	**<0.001***
	High AN PGS and high BMI PGS - Low AN PGS and high BMI PGS	0.029	0.044	0.654	0.91	0.067	0.056	1.194	0.63
**LMI**	Low AN PGS and low BMI PGS - High AN PGS and low BMI PGS	0.006	0.003	1.605	0.38	0.000	0.003	-0.008	**1**
	Low AN PGS and low BMI PGS - High AN PGS and high BMI PGS	-0.027	0.008	-3.415	**0.0036***	-0.032	0.008	-4.255	**<0.001***
	Low AN PGS and low BMI PGS - Low AN PGS and high BMI PGS	-0.033	0.004	-9.057	**<0.001***	-0.027	0.003	-7.892	**<0.001***
	High AN PGS and low BMI PGS - High AN PGS and high BMI PGS	-0.033	0.008	-3.913	**<0.001***	-0.032	0.008	-4.043	**<0.001***
	High AN PGS and low BMI PGS - Low AN PGS and high BMI PGS	-0.038	0.004	-8.514	**<0.001***	-0.027	0.004	-6.310	**<0.001***
	High AN PGS and high BMI PGS - Low AN PGS and high BMI PGS	-0.005	0.008	-0.631	0.92	0.005	0.008	0.602	0.93
**BMD**	Low AN PGS and low BMI PGS - High AN PGS and low BMI PGS	0.007	0.003	2.037	0.17	-0.003	0.003	-0.771	0.87
	Low AN PGS and low BMI PGS - High AN PGS and high BMI PGS	-0.021	0.008	-2.687	**0.04***	-0.018	0.007	-2.492	0.06
	Low AN PGS and low BMI PGS - Low AN PGS and high BMI PGS	-0.029	0.003	-8.308	**<0.001***	-0.023	0.003	-6.790	**<0.001***
	High AN PGS and low BMI PGS - High AN PGS and high BMI PGS	-0.027	0.008	-3.399	**0.0038***	-0.016	0.008	-2.032	0.17
	High AN PGS and low BMI PGS - Low AN PGS and high BMI PGS	-0.036	0.004	-8.243	**<0.001***	-0.021	0.004	-4.820	**<0.001***

	High AN PGS and high BMI PGS - Low AN PGS and high BMI PGS	-0.008	0.008	-0.997	0.75	-0.005	0.008	-0.603	0.93
**Weight**	Low AN PGS and low BMI PGS - High AN PGS and low BMI PGS	0.008	0.008	0.923	0.79	0.008	0.008	0.923	0.79
	Low AN PGS and low BMI PGS - High AN PGS and high BMI PGS	-0.096	0.019	-4.996	**<0.001***	-0.096	0.019	-4.996	**<0.001***
	Low AN PGS and low BMI PGS - Low AN PGS and high BMI PGS	-0.107	0.009	-12.340	**<0.001***	-0.107	0.009	-12.340	**<0.001***
	High AN PGS and low BMI PGS - High AN PGS and high BMI PGS	-0.104	0.020	-5.134	**<0.001***	-0.104	0.020	-5.134	**<0.001***
	High AN PGS and low BMI PGS - Low AN PGS and high BMI PGS	-0.115	0.011	-10.605	**<0.001***	-0.115	0.011	-10.605	**<0.001***
	High AN PGS and high BMI PGS - Low AN PGS and high BMI PGS	-0.011	0.020	-0.536	0.95	-0.011	0.020	-0.536	0.95
**Height**	Low AN PGS and low BMI PGS - High AN PGS and low BMI PGS	0.001	0.002	0.415	0.98	-0.001	0.002	-0.586	0.94
	Low AN PGS and low BMI PGS - High AN PGS and high BMI PGS	0.002	0.004	0.560	0.94	0.002	0.005	0.519	0.95
	Low AN PGS and low BMI PGS - Low AN PGS and high BMI PGS	-0.002	0.002	-0.754	0.87	-0.006	0.002	-2.819	**0.03***
	High AN PGS and low BMI PGS - High AN PGS and high BMI PGS	0.002	0.005	0.360	0.98	0.004	0.005	0.748	0.88
	High AN PGS and low BMI PGS - Low AN PGS and high BMI PGS	-0.002	0.002	-0.928	0.79	-0.005	0.003	-1.784	0.28
	High AN PGS and high BMI PGS - Low AN PGS and high BMI PGS	-0.004	0.005	-0.851	0.83	-0.009	0.005	-1.717	0.32

AN = anorexia nervosa; PGS = polygenic score; BMI = body mass index (weight in kilograms/height^2 in meters); FMI = fat mass index (fat mass in kilogram /height^2 in meters); LMI = lean mass index (lean mass in kilograms/height^2 in meters); BMD = bone mineral density (gram/cm^2).

[a] Full description of the Avon Longitudinal Study of Parents and Children is described elsewhere; [2–6].

[b] Categorical variable derived from dichotomizing the AN-PGS and the BMI-PGS. For both the AN and the BMI PGS, individuals with PGS scores at or greater than the 8th decile point were regarded as the "high PGS group" while those with scores lower were considered the "low PGS group". From the dichotomized AN-PGS and BMI-PGS we were able to create a categorical variable with two levels (1) high AN-PGS and high BMI-PGS (2) high AN-PGS and low BMI-PGS.

[c] Post-hoc comparisons corrected for multiple testing using Tukey's adjustment.

* Significant after accounting for multiple testing.

Supplementary material and methods

Genotyping quality control checks

Individuals with disproportionate levels of individual missingness (i.e., >3%), insufficient sample replication (identity by descent < 0.1), biological sex mismatch, and non-European ancestry (as defined by multi-dimensional scaling using the HapMap Phase II, release 22, reference populations) were excluded. Single nucleotide polymorphisms (SNPs) with a minor allele frequency (MAF) of < 1%, excessive missingness (i.e., call rate < 95%), or a departure from the Hardy–Weinberg equilibrium (P value < 5 x 10^{-7}) were removed. Imputation was conducted with Impute3 using the HRC 1.0 as the reference panel [1] and phasing was carried out using ShapeIT (v2.r644). Finally, post-imputation quality control checks were performed; any SNPs with MAF less than 1%, Impute3 information quality metric of < 0.8, and not confirming to Hardy-Weinberg equilibrium ($P < 5 \times 10^{-7}$) were removed. After data cleaning, a total of 8,654 individuals (4,225 females and 4,429 males) and 4,054,653 SNPs remained eligible for analyses.

Post-hoc power analysis

Power analysis were conducted using the polygenescore function in the AVENGEME package in R [8]. The following parameter were fixed for the power calculation of the anorexia nervosa PGS; number of independent SNPs = 46399; sample size training sample (sample size of the anorexia nervosa GWAS) = 72517 [9]; Proportion of variance explained by genetic effects in the training sample = 0.14 [9]; Proportion of markers with no effect on the training trait = 0.95; Estimated effect sizes are used as weights = TRUE; Training trait is binary = TRUE; Sampling = 0.31[9]; Effect sizes are to be shrunk to BLUPs = TRUE. The covariance between genetic effect sizes in the two samples (between anorexia nervosa and the trajectory

traits) were based on published genetic correlations for AN with BMI, FMI, LMI, BMD, weight, and height in the AN GWAS [9].

For the power calculation of the BMI PGS the following parameters were fixed: number of independent SNPs = 46399; sample size training sample = 700000; Proportion of variance explained by genetic effects in the training sample = 0.22; Proportion of markers with no effect on the training trait = 0.95; Estimated effect sizes are used as weights = TRUE; Training trait is binary = FALSE; Effect sizes are to be shrunk to BLUPs = TRUE. The covariance between genetic effect sizes in the two samples (between BMI and the trajectory traits) were either based on published genetic correlations for BMI with BMI[10], FMI, LMI, BMD [11], weight, and height or based on available online genetic correlations at https://ukbb-rg.hail.is/.

Supplemental references

1. McCarthy, S., Das, S., Kretzschmar, W., Delaneau, O., Wood, A.R., Teumer, A., Kang, H.M., Fuchsberger, C., Danecek, P., Sharp, K., et al. (2016). A reference panel of 64,976 haplotypes for genotype imputation. Nat. Genet. *48*, 1279–1283.

2. Fraser, A., Macdonald-wallis, C., Tilling, K., Boyd, A., Golding, J., Davey smith, G., Henderson, J., Macleod, J., Molloy, L., Ness, A., et al. (2013). Cohort profile: The avon longitudinal study of parents and children: ALSPAC mothers cohort. Int. J. Epidemiol. *42*, 97–110.

3. Boyd, A., Golding, J., Macleod, J., Lawlor, D.A., Fraser, A., Henderson, J., Molloy, L., Ness, A., Ring, S., and Smith, G.D. (2013). Cohort profile: The ’Children of the 90s’-The index offspring of the avon longitudinal study of parents and children. Int. J. Epidemiol. *42*, 111–127.

4. Golding, Pembrey, Jones, and The Alspac Study Team (2001). ALSPAC-The Avon Longitudinal Study of Parents and Children. Paediatr. Perinat. Epidemiol. *15*, 74–87.

5. Golding, J. (2004). The Avon Longitudinal Study of Parents and Children (ALSPAC)--study design and collaborative opportunities. Eur. J. Endocrinol. U119–U123.

6. Northstone, K., Lewcock, M., Groom, A., Boyd, A., Macleod, J., Timpson, N., and Wells, N. (2019). The Avon Longitudinal Study of Parents and Children (ALSPAC): an update on the enrolled sample of index children in 2019. Wellcome Open Res. *4*, 51.

7. Herle, M., Micali, N., Abdulkadir, M., Loos, R., Bryant-Waugh, R., Hübel, C., Bulik, C.M., and De Stavola, B.L. (2020). Identifying typical trajectories in longitudinal data: modelling strategies and interpretations. Eur. J. Epidemiol. *35*, 205–222.

8. Dudbridge, F. (2013). Power and Predictive Accuracy of Polygenic Risk Scores. PLoS Genet. *9*, e1003348.

9. Watson, H.J., Yilmaz, Z., Thornton, L.M., Hübel, C., Coleman, J.R.I.I., Gaspar, H.A., Bryois, J., Hinney, A., Leppä, V.M., Mattheisen, M., et al. (2019). Genome-wide association study identifies eight risk loci and implicates metabo-psychiatric origins for anorexia nervosa. Nat. Genet. *51*, 1207–1214.

10. Yengo, L., Sidorenko, J., Kemper, K.E., Zheng, Z., Wood, A.R., Weedon, M.N., Frayling, T.M., Hirschhorn, J., Yang, J., and Visscher, P.M. (2018). Meta-analysis of genome-wide association studies for height and body mass index in ∼700000 individuals of European ancestry. Hum. Mol. Genet. *27*, 3641–3649.

11. Liang, X., Wu, C., Zhao, H., Liu, L., Du, Y., Li, P., Wen, Y., Zhao, Y., Ding, M., Cheng, B., et al. (2018). Assessing the genetic correlations between early growth parameters and bone mineral density: A polygenic risk score analysis. Bone *116*, 301–306.
