# Supplementary material for: The impact of anorexia nervosa and BMI polygenic risk on childhood growth: A 20-year longitudinal population-based study
Source: Am J Hum Genet. 2022 Jun 14;109(7):1242–54. doi: 10.1016/j.ajhg.2022.05.005 (PMC9300758; doi:10.1016/j.ajhg.2022.05.005)
Supplement: Document S2. Article plus supplemental information [file mmc2.pdf]

The impact of anorexia nervosa and BMI polygenic risk on childhood growth: A 20-year longitudinal population-based study

Authors

Mohamed Abdulkadir, Christopher Hübel, Moritz Herle, Ruth J.F. Loos, Gerome Breen, Cynthia M. Bulik, Nadia Micali

Correspondence

nadia.micali@hcuge.ch

Abdulkadir et al., 2022, The American Journal of Human Genetics *109*, 1242–1254
July 7, 2022 © 2022 The Authors.
https://doi.org/10.1016/j.ajhg.2022.05.005

CellPress

The impact of anorexia nervosa and BMI polygenic risk on childhood growth: A 20-year longitudinal population-based study

Mohamed Abdulkadir,[1,2,5,14] Christopher Hübel,[3,4,5,6,14] Moritz Herle,[8,14] Ruth J.F. Loos,[9,10] Gerome Breen,[3,4] Cynthia M. Bulik,[6,11,12,14] and Nadia Micali[1,2,7,13,14,*]

Summary

Growth deviating from the norm during childhood has been associated with anorexia nervosa (AN) and obesity later in life. In this study, we examined whether polygenic scores (PGSs) for AN and BMI are associated with growth trajectories spanning the first two decades of life. AN PGSs and BMI PGSs were calculated for participants of the Avon Longitudinal Study of Parents and Children (ALSPAC; n = 8,654). Using generalized (mixed) linear models, we associated PGSs with trajectories of weight, height, body mass index (BMI), fat mass index (FMI), lean mass index (LMI), and bone mineral density (BMD). Female participants with AN PGSs one standard deviation (SD) higher had, on average, 0.004% slower growth in BMI between the ages 6.5 and 24 years and a 0.4% slower gain in BMD between the ages 10 and 24 years. Higher BMI PGSs were associated with faster growth for BMI, FMI, LMI, BMD, and weight trajectories in both sexes throughout childhood. Female participants with both a high AN PGS and a low BMI PGS showed slower growth compared to those with both a low AN PGS and a low BMI PGS. We conclude that AN PGSs and BMI PGSs have detectable sex-specific effects on growth trajectories. Female participants with a high AN PGS and low BMI PGS likely constitute a high-risk group for AN, as their growth was slower compared to their peers with high PGSs on both traits. Further research is needed to better understand how the AN PGS and the BMI PGS co-influence growth during childhood and whether a high BMI PGS can mitigate the effects of a high AN PGS.

Introduction

Anorexia nervosa (AN) is a serious psychiatric disorder that is characterized by low fat and lean mass.[1–3] Observations from genome-wide association studies (GWASs) suggest that genomic variants that influence body composition are also associated with psychiatric traits.[4] Genetically, AN is negatively correlated with body mass index (BMI), fat mass, fat-free mass, and obesity,[4] suggesting that biological mechanisms contributing to AN may also influence body composition. This association is supported by several studies showing that low premorbid BMI is associated with AN in adolescence.[5,6] Furthermore, an Avon Longitudinal Study of Parents and Children (ALSPAC) study reported that individuals who go on to develop AN followed lower BMI trajectories (as early as age 2 years) compared to their peers that did not develop an eating disorder (ED).[7]

In contrast to low body weight, high body weight has not only been associated with increased risk for cardiovascular disease but also with psychiatric disorders (e.g., mood disorders and anxiety disorders).[8,9] In addition, individuals with high body weight face stigmatization and discrimination from the public and health professionals, which can exacerbate the negative health effects of obesity.[10–12] Similar to AN, BMI has been extensively studied on a genetic level and is a heritable polygenic trait.[13,14] Khera et al.[15] reported that a BMI polygenic score (PGS), calculated by summing the BMI-increasing alleles of all variants of a BMI GWAS, weighted by their reported effect sizes,[13] is associated with body weight at different time points during childhood and adolescence. For example, individuals with a BMI PGS in the top decile have modest yet significantly higher birth weight (+60 g) than individuals with a BMI PGS in the bottom decile.[15] However, this difference in weight increases over time, reaching 3.5 kg by age 8 years and 12.3 kg by age 18 years.[15] These findings highlight differences in growth associated with the polygenic liability to high BMI.

In summary, both AN and BMI have a genetic component that can be summarized by PGSs, and these genetic components are inversely correlated. However, it is unclear how genetic risk for both traits, individually and

[1]Department of Pediatrics Gynaecology and Obstetrics, Faculty of Medicine, University of Geneva, Geneva, Switzerland; [2]Department of Psychiatry, Faculty of Medicine, University of Geneva, Geneva, Switzerland; [3]Social, Genetic & Developmental Psychiatry Centre, Institute of Psychiatry, Psychology & Neuroscience, King's College London, London, UK; [4]UK National Institute for Health Research (NIHR) Biomedical Research Centre for Mental Health, South London and Maudsley Hospital, London, UK; [5]National Centre for Register-based Research, Aarhus Business and Social Sciences, Aarhus University, Aarhus, Denmark; [6]Department of Medical Epidemiology and Biostatistics, Karolinska Institutet, Stockholm, Sweden; [7]Great Ormond Street Institute of Child Health, University College London, London, UK; [8]Department of Biostatistics & Health Informatics, Institute of Psychiatry, Psychology & Neuroscience, King's College London, London, UK; [9]Novo Nordisk Foundation Center for Basic Metabolic Research, Department of Health and Medical Sciences, University of Copenhagen, Copenhagen, Denmark; [10]Icahn School of Medicine at Mount Sinai, New York, NY, USA; [11]Department of Psychiatry, University of North Carolina at Chapel Hill, Chapel Hill, NC, USA; [12]Department of Nutrition, University of North Carolina at Chapel Hill, Chapel Hill, NC, USA; [13]Mental Health Services in the Capital Region of Denmark, Eating Disorders Research Unit, Psychiatric Centre Ballerup, Ballerup, Denmark
[14]These authors contributed equally
*Correspondence: nadia.micali@hcuge.ch
https://doi.org/10.1016/j.ajhg.2022.05.005.
© 2022 The Authors. This is an open access article under the CC BY license (http://creativecommons.org/licenses/by/4.0/).

combined, affect growth developmentally during the first two decades of life. We identified individuals considered to be at high risk (defined as PGS in the top two deciles) for either AN or BMI and compared them with their peers with lower risk (defined as PGS in the lower 8 deciles) for the same trait. We then studied the longitudinal effects of the AN PGS[4] and the BMI PGS[14] (separately and combined) on weight, height, BMI, fat mass index (FMI), lean mass index (LMI), and bone mineral density (BMD) growth trajectories during the first two decades of life using data from ALSPAC.[16–20] We hypothesized that a higher AN PGS would be associated with slower growth for the weight, BMI, FMI, LMI, and BMD trajectories. Previous studies reported no genetic correlation between height and AN, and therefore we used a height trajectory as a negative control.[3] We also hypothesized that a higher BMI PGS would be associated with faster growth trajectories. Lastly, we hypothesized that individuals with both a high AN PGS and low BMI PGS would represent a subgroup at higher risk for poor growth (slower growth) compared to those with both a low AN PGS and a low BMI PGS.

Material and methods

Participants

The ALSPAC study is an ongoing population-based birth-cohort study of 14,541 mothers and their children (that were born between April 1, 1991 and December 31, 1992) residing in the southwest of England (UK).[16–20] From the 15,541 pregnancies, 13,988 were alive at 1 year. At age 7 years, this sample was bolstered with an additional 913 children. The total sample size for analyses using any data collected after the age of 7 is therefore 15,454 pregnancies; of these, 14,901 were alive at 1 year of age. Participants are assessed at regular intervals using clinical interviews, self-report questionnaires, medical records, and physical examinations. Study data were collected and managed using REDCap (Research Electronic Data Capture) electronic data capture tools hosted at University of Bristol.[21,22] REDCap is a secure, web-based software platform designed to support data capture for research studies, providing (1) an intuitive interface for validated data capture, (2) audit trails for tracking data manipulation and export procedures, (3) automated export procedures for seamless data downloads to common statistical packages, and (4) procedures for data integration and interoperability with external sources. Please note that the study website contains details of all data that are available and includes a fully searchable data dictionary and variable search tool: http://www.bristol.ac.uk/alspac/researchers/our-data/. To avoid potential confounding due to relatedness, one sibling per set of multiple births was randomly selected to guarantee independence of participants; this resulted in the removal of 75 individuals. Furthermore, individuals who are closely related to each other, defined as a phi hat >0.2 (calculated using PLINK v1.90b), were removed; specifically, we removed any duplicates or monozygotic twins, first-degree relatives (parent-offspring and full siblings), and second-degree relatives (half siblings, uncles, aunts, grandparents, and double cousins). The authors assert that all procedures contributing to this work comply with the ethical standards of the relevant national and institutional committees on human experimentation and with the Helsinki Declaration of 1975, as revised in 2008. Ethical approval for the study was obtained from the ALSPAC Ethics and Law Committee and the Local Research Ethics Committees. Informed consent for the use of data collected via questionnaires and clinics was obtained from participants following the recommendations of the ALSPAC Ethics and Law Committee at the time. The main caregiver initially provided consent for child participation, and from the age 16 years the offspring themselves have provided informed written consent.

Measures

Weight, height, and BMI

Numerous measurements of weight and height were collected from different sources (i.e., routine clinic visits, information collected from midwives, linkage to child health records) between birth and age 24 years. Information on weight was collected at research clinic visits annually up to age 14 years and further clinic measurements at ages 16, 18, and 24 years using the Tanita Body Fat Analyzer (Tanita TBFUK Ltd.) to the nearest 50 g. During the same clinic visits, height (standing) was measured to the nearest millimeter with shoes and socks removed using a Holtain stadiometer (Holtain Ltd, Crymych, Pembs, UK). The different measurements of weight and height were highly correlated (Figure S1) across various methods.[16,23] Information on child and adolescent BMI (weight in kilograms/height squared in meters) was derived using weight and height measurements obtained during clinic visits.

FMI, LMI, and BMD

All ALSPAC participants were invited to undergo whole-body dual-energy X-ray absorptiometry (DEXA) scans using the Lunar Prodigy DEXA scanner as part of face-to-face visits at the ages of 10, 12, 14, 16, 18, and 24 years. FMI was calculated by dividing total body fat mass (in kilograms) by height (in meters) squared. Similarly, LMI was calculated by dividing total lean mass by height (in meters) squared. Additionally, whole-body (minus head) BMD was also estimated using the Lunar Prodigy DEXA scanner.

Trajectory modeling

Censoring for the presence of an ED

To derive the trajectories for each outcome, we censored for the presence of any ED, i.e., AN, bulimia nervosa, and binge-eating disorder. Information on a probable ED was available at ages 14, 16, and 18 years (see Micali et al.[24] and Hübel et al.[25] for more information on how ED diagnoses were derived). The presence of an ED diagnosis at age 14 years meant that all values for that individual regarding their measurement (BMI, FMI, LMI, etc.) at age 14 years up to age 24 years were set to missing. This was also done for the presence of an ED diagnosis at age 16 years (set values at age 16 and beyond to missing) and 18 years (set values at age 18 and beyond to missing). Therefore, censoring did not lead to loss of participants in the analyses, but rather loss of observations ($n = 1{,}055$). This censoring allowed us to derive unbiased results in the following longitudinal modeling, while retaining the largest amount of data possible. This is important, as these models are sensitive to outliers and including individuals with EDs would likely introduce extreme values in the distribution.

Spline modeling

To capture the potential impact of AN on growth, we derived a BMI trajectory across both childhood and adolescence for all participants jointly. Prior to analyses, BMI values (Table S1 and

Figure S2) were transformed using the natural log due to the right-skewed distribution of the data. Spline models involve placing spline points (knots) at time points where the direction of growth changes. This is necessary as children's growth in the first two decades of life is not linear, but follows a more complex pattern, rendering standard growth models unsuitable to accurately reflect the data.[26] The advantage of linear spline models is that they allow knot points to be fitted at different ages to derive periods of change (between the knots) that are approximately linear. After visual inspection of the BMI medians at each time point, two spline points (knots), in addition to the starting point (intercept) at 4 months and the final data wave (24 years), were placed, creating the following periods of linear growth: between ages 4 months and 1 year, between ages 1 and 6.5 years, and between ages 6.5 and 24 years. Mixed-effects spline models were run to describe the longitudinal growth outcomes. The mixed-effects framework lends itself to the analyses of repeated measures as it accounts for the non-independence of measures within an individual. After model fitting, we extracted parameters of slopes using the best linear unbiased predictions (BLUPs). The linear spline modeling resulted in three slopes (coefficients), which correspond with the slopes in the periods of growth between age 4 months and 1 year, growth between age 1 year and 6.5 years, and growth between age 6.5 years and 24 years. Spline models were obtained using STATA (v.15).

Genotyping

Genotype data were available for 9,915 out of the total of 15,247 ALSPAC participants. Participants were genome-wide genotyped on the Illumina HumanHap550 quad chip. Following quality control of the genetic data, a total of 8,654 participants with genotyping data and at least one outcome measure were included in the analyses (Table 1). Details of the quality control checks are described in the supplemental information.

PGS calculations

The AN PGS was derived from the GWAS on AN by the Eating Disorders Working Group of the Psychiatric Genomics Consortium (PGC-ED; $n_{cases} = 16,992$, $n_{controls} = 55,525$),[4] and the BMI PGS was derived from the BMI GWAS conducted by the Genetic Investigation of Anthropometric Traits (GIANT; n ~700,000 individuals).[14] The datasets will be referred to as the AN and BMI discovery cohorts, respectively. The PGSs were calculated using the polygenic risk score continuous shrinkage (PRS-CS) software; a method that infers posterior SNP effect sizes under continuous shrinkage (CS) priors.[27]

Furthermore, we derived a categorical variable using the AN PGS and BMI PGS as follows. We dichotomized the AN and the BMI PGS based on a cut-off value at the 8th decile of the PGS distribution. This value was selected based on previous studies that reported that individuals in top deciles of BMI PGS and schizophrenia PGS are at greater risk than those in lower deciles for being overweight and being diagnosed with schizophrenia, respectively.[15,28] We did not choose the highest decile as cut-off, as this would have resulted in a small sample size and thus low statistical power for this particular bin. Individuals with a PGS lower than the 8th decile were grouped into a "low PGS" group, and those with a PGS score at or higher than 8th decile were grouped into a "high PGS" group. Based on this grouping, we were able to determine four categories: individuals with a (1) low PGS for AN and low PGS for BMI, (2) high PGS for AN and low PGS for BMI, (3) low PGS for AN and high PGS for BMI, and (4) high PGS for AN and high PGS for BMI. The "low AN PGS and low BMI PGS" group was used as the reference category in the analyses.

Statistical analyses

PGS analyses

In the first set of analyses, we addressed whether the AN PGS and BMI PGS separately were associated with body composition trajectories. For the BMI spline trajectory, we regressed each derived slope onto the standardized AN PGS or the BMI PGS and the first four ancestry-informative principal components. Data for FMI, LMI, and BMD were measured objectively at 10, 12, 14, 16, 18, and 24 years during face-to-face visits, as described above. For uniformity, we restricted analyses of weight and height to measurements at these ages as growth is more linear from mid-childhood onwards. Height was included to test the quality of methodological approach as a negative control for the association with the AN PGS.[3] FMI, LMI, BMD, weight, and height trajectories were analyzed using linear mixed-effects regression (LMER) using the LMER function from the LME4 package in R;[29] the standardized AN PGS or BMI PGS, the first four ancestry-informative principal components, and age were added as fixed effects. For the linear mixed-effects models, the intercept and the slope were all allowed to vary randomly across individuals. We stratified our analyses on biological sex; phenotypic sex differences in body composition have been reported in the general population that are detectable as early as adolescence.[30,31] For comparisons between females and males, we tested whether the slopes of the association of the AN PGS or the BMI PGS with the body composition trajectories differed using the methods described by Clogg et al. and Paternoster et al.[32,33] Considering that the BMI slopes are highly correlated, we additionally corrected for slope(s) that preceded the one that is being analyzed; i.e., for the model in which the slope between the ages 1 year and 6.5 years is the outcome, we controlled for the slope preceding this period (slope between the ages 4 months and 1 year), and for the model in which the slope between the ages 6.5 years and 24 years is the outcome, we controlled for the slopes preceding this period (slope between the ages 4 months and 1 year and slope between ages 1 year and 6.5 years). We report for each model the beta (as a measure of effect size, with 95% confidence intervals) and the percent change in the estimated parameter to ease the interpretation of the beta point estimates.

Correction for multiple testing across all tests ($n = 648$) was done by calculating false discovery rate-corrected Q values.[34] The significance threshold was met if the false discovery rate-adjusted Q was <0.05.

Extreme group comparison of the PGS analyses

The effects of PGS are often most discernable in the most extreme deciles. We therefore tested whether groups characterized by a particularly high PGS load differ from one another. Note that this differs from testing a formal interaction between the AN PGS and the BMI PGS, as our focus is on understanding the difference in growth in the extreme end of the PGS distribution. In this second set of analyses, we used LMER to determine the association between the body composition measures (BMI, FMI, LMI, BMD, weight, and height) and the derived categorical AN and BMI PGS (see PGS calculations). For each regression model, the derived categorical variable of the AN PGS or BMI PGS, age, and the first four ancestry-informative principal components were included as fixed effects. We included random intercepts and slope for each individual in the model to account for variance in body composition

Table 1. Descriptive data from the Avon Longitudinal Study of Parents and Children (ALSPAC)

Body composition measure	Age (years)	Female		Male	
		n	Median (IQR)	n	Median (IQR)
BMI (kg/m^2)[a]	10	3,072	17.31 (15.77, 19.41)	3,026	16.77 (15.61, 18.73)
	12	2,902	18.6 (16.74, 21.14)	2,797	17.96 (16.43, 20.5)
	14	2,433	20.17 (18.43, 22.58)	2,394	19.28 (17.71, 21.45)
	16	1,496	20.95 (19.47, 23.14)	1,289	20.83 (19.14, 22.78)
	18	1,986	22.14 (20.35, 24.78)	1,681	21.76 (20.08, 24.34)
	24	1,671	23.63 (21.47, 27.04)	1,131	24.25 (21.97, 27.09)
FMI (kg/m^2)	10	2,932	4.4 (3.14, 6.18)	2,880	2.96 (2.08, 4.67)
	12	2,862	4.91 (3.52, 7.11)	2,750	3.65 (2.53, 5.9)
	14	2,406	5.77 (4.28, 7.74)	2,353	3.11 (2.09, 5.26)
	16	1,266	6.5 (5.00, 8.36)	1,066	2.67 (1.83, 4.14)
	18	1,895	7.13 (5.68, 9.28)	1,624	3.37 (2.22, 5.64)
	24	1,618	8.07 (6.43, 10.61)	1,100	5.7 (4.32, 7.8)
LMI (kg/m^2)	10	2,932	12.07 (11.5, 12.69)	2,880	12.98 (12.43, 13.55)
	12	2,862	12.64 (11.95, 13.42)	2,750	13.28 (12.63, 14)
	14	2,406	13.39 (12.7, 14.1)	2,353	14.87 (13.9, 15.92)
	16	1,266	13.56 (12.81, 14.3)	1,066	15.93 (14.76, 16.98)
	18	1,895	13.86 (13.17, 14.64)	1,624	17.19 (16.16, 18.18)
	24	1,618	14.8 (13.95, 15.82)	1,100	17.45 (16.25, 18.83)
BMD (g/cm^2)	10	2,965	0.77 (0.74, 0.81)	2,900	0.78 (0.75, 0.82)
	12	2,865	0.85 (0.8, 0.9)	2,756	0.84 (0.80, 0.89)
	14	2,406	0.96 (0.91, 1.01)	2,354	0.95 (0.90, 1.01)
	16	2,050	1 (0.96, 1.05)	1,952	1.06 (0.99, 1.12)
	18	1,902	1.04 (0.99, 1.09)	1,634	1.14 (1.08, 1.21)
	24	1,619	1.19 (1.13, 1.26)	1,101	1.32 (1.23, 1.39)
Weight (kg)	10	3,105	33.6 (29.6, 38.8)	3,045	33 (29.4, 37.8)
	12	2,904	42.8 (37, 50.2)	2,801	40.6 (35.8, 47.6)
	14	2,433	53.4 (47.8, 60.2)	2,394	53 (46.8, 61.35)
	16	1,599	57 (52, 64)	1,330	66 (59, 74)
	18	1,986	61 (55.1, 68.4)	1,683	70.4 (63.7, 79)
	24	1,671	64.9 (58.7, 75.55)	1,131	79 (70.7, 88.5)
Height (cm)	10	3,074	139.05 (135, 143.4)	3,027	139.8 (135.7, 143.9)
	12	2,904	151.4 (146.4, 156.2)	2,797	149.8 (145.1, 154.7)
	14	2,438	162.1 (157.8, 166.3)	2,394	165.2 (158.9, 170.8)
	16	1,666	165 (160, 170)	1,361	178 (173, 183)
	18	1,988	165.1 (161.1, 169.2)	1,683	179 (174.45, 183.4)
	24	1,672	165.8 (161.88, 170)	1,131	180 (175.5, 184.5)

Data are presented on age, BMI, FMI, LMI, weight, and height. The sample size per outcome varies. Observations were censored for the presence of an eating disorder (ED), i.e., anorexia nervosa, bulimia nervosa, and binge-eating disorder. Information on a probable ED was available at age 14, 16, and 18 years.[24,25] The presence of an ED diagnosis at age 14 years meant that all values for that individual regarding their measurement (BMI, FMI, LMI, etc.) at age 14 years up to age 24 years were set to missing. This was also done for the presence of an ED diagnosis at age 16 years (set values at age 16 and beyond to missing) and 18 years (set values at age 18 and beyond to missing). BMI was calculated using objectively measured weight and height during a routine clinic visit at age 24 years. Height was measured to the nearest millimeter using a Harpenden Stadiometer (Holtain Ltd.), and weight was measured using the Tanita body fat analyzer (Tanita TBF UK Ltd.) to the nearest 50 grams. FMI, LMI, and BMD were derived using a Lunar Prodigy dual-energy X-ray absorptiometry (DEXA) scanner (GE Medical Systems Lunar, Madison, WI, USA). FMI and LMI were calculated by dividing each measure (in kilograms) by height squared (in meters). BMD was calculated for the whole body excluding the head values. IQR, interquartile range; BMI, body mass index (weight in kilograms/height^2 in meters); FMI, fat mass index (fat mass in kilograms/height^2 in meters); LMI, lean mass index (lean mass in kilograms/height^2 in meters); BMD, bone mineral density.
[a]To limit the size of this table, only the BMI values at age 10 years and later are shown; for BMI values prior to age 10 years, see Table S1.

Table 2. Associations of the AN PGS with body composition stratified for biological sex in ALSPAC

Trajectory	Parameter	Female			Male		
		Beta[a]	% change[b]	Q[c]	Beta[a]	% change[b]	Q[c]
BMI	Slope age 4 months to 1 year	1.0000 (1.000, 1.0000)	<0.001	0.76	1.0000 (1.0000, 1.0000)	<0.001	0.71
BMI	Slope age 1–6.5 years	1.0000 (1.0000, 1.0001)	<0.001	0.36	1.0000 (1.0000, 1.0000)	< −0.001	0.74
BMI	Slope age 6.5–24 years	1.0000 (0.9999, 1.0000)	−0.004	<0.001[d]	1.0000 (1.0000, 1.0000)	< −0.001	0.06
FMI	Slope age 10–24 years	0.9919 (0.9774, 1.0067)	−0.81	0.36	0.9942 (0.9758, 1.0130)	−0.58	0.62
LMI	Slope age 10–24 years	0.9973 (0.9944, 1.0002)	−0.27	0.11	0.9993 (0.9961, 1.0027)	−0.07	0.74
BMD	Slope age 10–24 years	0.9960 (0.9929, 0.9991)	−0.40	0.02[d]	0.9986 (0.9950, 1.0022)	−0.14	0.51
Weight	Slope age 10–24 years	0.9937 (0.9867, 1.0008)	−0.63	0.13	0.9956 (0.9877, 1.0036)	−0.44	0.36
Height	Slope age 10–24 years	0.9990 (0.9974, 1.0006)	−0.10	0.31	0.9991 (0.9970, 1.0012)	−0.09	0.48

Full description of ALSPAC is provided elsewhere.[16–20] BMI trajectory was derived using spline modeling. Prior to deriving the trajectory, BMI was transformed using the natural logarithm. For the spline modeling, three spline segments (slopes) were created: a slope capturing growth between age 4 months and 1 year, a slope capturing growth between age 1 and 6.5 years, and a slope capturing growth between age 6.5 and 24 years. FMI, LMI, BMD, weight, and height trajectories were analyzed using generalized linear mixed models.[35] The AN PGS and the first four ancestry-informative principal components were added as fixed effects. The intercept and the slope were all allowed to vary randomly across individuals. BMI, body mass index (weight in kilograms/height^2 in meters); FMI, fat mass index (fat mass in kilograms/height^2 in meters); LMI, lean mass index (lean mass in kilograms/height^2 in meters); BMD, bone mineral density (gram/cm^2).
[a]Betas reflect one SD change in the standardized (to mean zero and SD of one) AN PGS.
[b]Considering that the outcomes were log-transformed, we report the percent change in the outcome for one SD increase in the PGS to ease the interpretation of the betas.
[c]Benjamini & Hochberg false discovery rate adjustment for the number tests performed.[34]
[d]Significant after accounting for multiple testing using the false discovery rate-corrected Q values. Significance was set at Q < 0.05.

measures due to inter-individual differences. The analyses were stratified by sex, given differences in body composition.[31] The "low AN PGS and low BMI PGS" group was used as the reference category in the analyses. For this set of tests, correction for multiple testing was done by calculating false discovery rate-corrected Q values.[34] The significance threshold was met if the false discovery rate-adjusted Q was <0.05.

Post-hoc analyses of the extreme group comparisons

Based on the reported negative genetic correlations between AN and BMI,[4] we also examined whether the BMI PGS mitigates the effect of the AN PGS. Therefore, we carried out post-hoc analyses, comparing the above extreme groups in order to understand how they differed from one another. Post-hoc comparisons were corrected for multiple testing using Tukey's adjustment.

Results

Sample description

Following quality control of the genetic data, a total of 8,654 children with genotyping data and at least one outcome measure were included in the analyses (Table 1, Table S1, and Figure S2).

Association of the AN PGS with growth trajectories

We observed several significant associations between the AN PGS and growth trajectories (on the additive scale) exclusively in the periods of linear growth in female participants (Table 2). For female participants, between the ages 6.5 and 24 years, a one-SD increase in the AN PGS was associated with a 0.004% slower growth per year in their BMI trajectory, given their BMI at 4 months (the intercept).

Furthermore, in female participants, on average a one-SD-higher AN PGS was associated with a 0.40% slower gain per year for their BMD trajectory between the ages 10 and 24 years, given BMD at 10 years. There was no significant association between the AN PGS and growth trajectories in male participants.

We did not observe a significant difference in slopes of associations between the AN PGS and growth trajectories between females and males (Table S2). Post-hoc power analysis indicates that the AN PGS analyses were well powered (power >0.8; post-hoc power analysis supplementary note and Table S3)

Association of the BMI PGS with growth trajectories

The BMI PGS was associated with periods of linear growth of the BMI trajectory between the ages 1 and 24 years; a one-SD-higher BMI PGS was associated with a 0.02% faster growth in BMI per year in female and male participants (Table 3).

Between ages 10 and 24 years, a one-SD-higher BMI PGS was associated with a faster growth/gain in FMI (16.74%), LMI (2.11%), BMD (1.64%), and weight (6.42%) in female participants. We observed no association between the BMI PGS and the height trajectory in females. We observed a similar pattern of results in male participants, with a notable exception for the FMI trajectory, in which a one-SD-higher BMI PGS was associated with a 20.4% faster growth in FMI between ages 10 and 24 years.

Furthermore, slopes of associations of the BMI PGS with body composition trajectories differed between females and males. Increases in BMI were more pronounced in females, a 20% steeper slope, in the association between the BMI PGS and the BMI trajectory between ages 4 months and 1 year compared to males (Table S4). Higher BMI PGS corresponded with increases in FMI in both sexes; however, this increase in FMI was more pronounced in males

Table 3. Associations of the BMI PGS with body composition stratified for biological sex in ALSPAC

Trajectory	Parameter	Female			Male		
		Beta[a]	% change[b]	Q[c]	Beta[a]	% change[b]	Q[c]
BMI	Slope age 4 months to 1 year	1.0000 (1.0000, 1.0000)	<0.001	<0.001[d]	1.0000 (1.0000, 1.0000)	<0.001	<0.001[d]
BMI	Slope age 1–6.5 years	1.0002 (1.0001, 1.0002)	0.02	<0.001[d]	1.0002 (1.0002, 1.0002)	0.02	<0.001[d]
BMI	Slope age 6.5–24 years	1.0002 (1.0002, 1.0002)	0.02	<0.001[d]	1.0002 (1.0001, 1.0002)	0.02	<0.001[d]
FMI	Slope age 10–24 years	1.1673 (1.1516, 1.1832)	16.73	<0.001[d]	1.2040 (1.1837, 1.2247)	20.4	<0.001[d]
LMI	Slope age 10–24 years	1.0211 (1.0183, 1.0239)	2.11	<0.001[d]	1.0185 (1.0153, 1.0217)	1.85	<0.001[d]
BMD	Slope age 10–24 years	1.0164 (1.0133, 1.0195)	1.64	<0.001[d]	1.0142 (1.0107, 1.0178)	1.42	<0.001[d]
Weight	Slope age 10–24 years	1.0642 (1.0572, 1.0713)	6.42	<0.001[d]	1.0584 (1.0506, 1.0663)	5.84	<0.001[d]
Height	Slope age 10–24 years	1.0016 (1.0000, 1.0033)	0.16	0.09	1.0023 (1.0003, 1.0044)	0.23	0.05

Full description of ALSPAC is provided elsewhere.[16–20] BMI trajectory was derived using spline modeling. Prior to deriving the trajectory, BMI was transformed using the natural logarithm. For the spline modeling, three spline segments (slopes) were created: a slope capturing growth between age 4 months and 1 year, a slope capturing growth between age 1 and 6.5 years, and a slope capturing growth between age 6.5 and 24 years. FMI, LMI, BMD, weight, and height trajectories were analyzed using generalized linear mixed models.[35] The BMI PGS and the first four ancestry-informative principal components were added as fixed effects. The intercept and the slope were all allowed to vary randomly across individuals. BMI, body mass index (weight in kilograms/height^2 in meters); FMI, fat mass index (fat mass in kilograms/height^2 in meters); LMI, lean mass index (lean mass in kilograms/height^2 in meters); BMD, bone mineral density (gram/cm^2).
[a]Betas reflect one SD change in the standardized (to mean zero and SD of one) BMI PGS.
[b]Considering that the outcomes were log-transformed we report the percent change in the outcome for one SD increase in the PGS to ease the interpretation of the betas.
[c]Benjamini & Hochberg false discovery rate adjustment for the number of tests performed.[34]
[d]Significant after accounting for multiple testing using the false discovery rate-corrected Q values. Significance was set at $Q < 0.05$.

than in females—the slope of this association was 20% steeper in males than in females. Post-hoc power analysis indicates that the BMI PGS analyses were well powered (power >0.8; post-hoc power analysis supplementary note and Table S3).

Extreme group comparisons

The effects of PGS are often most discernable in the most extreme deciles, so we therefore tested whether groups characterized by a particularly high PGS load differed from one another. Note that this differs from testing a formal interaction between the AN PGS and the BMI PGS, as our focus is on understanding difference in growth at the extreme end of the PGS distribution. For the extreme group comparisons, for the BMI trajectory, we focused only on the period of growth between ages 6.5 and 24 years as the AN PGS was associated only with this stage of growth (see Table 2). For the FMI, LMI, BMD, weight, and height trajectories, we analyzed the period between ages 10 and 24 years. Sample sizes of the extreme group comparisons can be found in Table S5.

Individuals with a low AN PGS and a high BMI PGS had on average faster growth compared to individuals with a low AN PGS and a low BMI PGS. The difference was most pronounced for the FMI trajectory (Table 4). Female participants with a low AN PGS and a high BMI PGS had a 27.81% faster growth in FMI compared to their peers with a low AN PGS and a low BMI PGS. Male participants with a low AN PGS and a high BMI PGS also showed faster growth in FMI (38.14%) compared to their peers with a low AN PGS and a low BMI PGS. In addition, male participants with a low AN PGS and a high BMI PGS showed 0.62% faster growth in height compared to the reference group. Furthermore, although not statistically significant, we observed a trend in which female participants with a high AN PGS and a low BMI PGS had slower growth/gain for the BMI, FMI, LMI, BMD, weight, and height trajectories compared to the reference category of individuals with a low AN PGS and a low BMI PGS (Figure 1 and Table 4).

Post-hoc analyses of extreme group comparisons

In order to understand how the various PGS groups differ from one another, we conducted post-hoc analyses of the extreme group comparisons. Though the group with high AN PGS and low BMI PGS did not differ from the reference category (Table 4), this group did differ (slower growth) significantly from the group with high AN PGS and high BMI PGS and from the group with low AN PGS and high BMI PGS (Table 5 and S6).

Discussion

Using longitudinal data across the first two decades of life, we report that common genomic variants associated with AN and BMI are significantly associated with growth trajectories. Female participants with a high AN genetic liability differ significantly in growth, as marked by slower growth as early as age 6.5 years for the BMI trajectory and age 10 years for the BMD trajectory. This effect was not observed in male participants.

Sex differences in body composition have been well documented in the medical literature, with men on average being taller and having more lean body mass, higher BMD, and lower fat mass compared to women.[36] These biological differences are driven by both

Table 4. **Associations of the combined AN and BMI PGS with the body composition measures stratified for biological sex using linear mixed models in ALSPAC**

		Female			Male		
**Trajectory**	**PGS category**	**Beta**[a]	**% change**[b]	**Q**[c]	**Beta**[a]	**% change**[b]	**Q**[c]
BMI (age 6.5–24 years)	Low AN and high BMI	1.10 (1.08, 1.11)	9.78	<0.001[d]	1.07 (1.06, 1.09)	7.41	<0.001[d]
BMI (age 6.5–24 years)	High AN and high BMI	1.11 (1.08, 1.14)	10.74	<0.001[d]	1.10 (1.08, 1.13)	10.28	<0.001[d]
BMI (age 6.5–24 years)	High AN and low BMI	1.10 (0.99, 1.01)	−0.17	0.89	1.00 (0.99, 1.01)	0.09	0.93
FMI (age 10–24 years)	Low AN and high BMI	1.28 (1.23, 1.33)	27.81	<0.001[d]	1.38 (1.32, 1.45)	38.14	<0.001[d]
FMI (age 10–24 years)	High AN and high BMI	1.32 (1.21, 1.43)	31.56	<0.001[d]	1.48 (1.33, 1.64)	47.76	<0.001[d]
FMI (age 10–24 years)	High AN and low BMI	0.99 (0.95, 1.02)	−1.29	0.65	1.02 (0.97, 1.07)	1.51	0.71
LMI (age 10–24 years)	Low AN and high BMI	1.03 (1.03, 1.04)	3.32	<0.001[d]	1.03 (1.02, 1.03)	2.77	<0.001[d]
LMI (age 10–24 years)	High AN and high BMI	1.03 (1.01, 1.04)	2.77	0.001[d]	1.03 (1.02, 1.05)	3.26	<0.001[d]
LMI (age 10–24 years)	High AN and low BMI	0.99 (0.99, 1.00)	−0.56	0.17	1.00 (0.99, 1.01)	0.00	0.99
BMD (age 10–24 years)	Low AN and high BMI	1.03 (1.02, 1.04)	2.92	<0.001[d]	1.03 (1.02, 1.03)	2.77	<0.001[d]
BMD (age 10–24 years)	High AN and high BMI	1.02 (1.01, 1.04)	2.09	0.012[d]	1.03 (1.02, 1.05)	3.26	<0.001[d]
BMD (age 10–24 years)	High AN and low BMI	0.99 (0.99, 1.00)	−0.68	0.07	1.00 (0.99, 1.01)	0.00	0.99
Weight (age 10–24 years)	Low AN and high BMI	1.11 (1.09, 1.13)	11.29	<0.001[d]	1.12 (1.10, 1.13)	11.52	<0.001[d]
Weight (age 10–24 years)	High AN and high BMI	1.10 (1.06, 1.14)	10.08	<0.001[d]	1.12 (1.08, 1.16)	11.85	<0.001[d]
Weight (age 10–24 years)	High AN and low BMI	0.99 (0.98, 1.01)	−0.77	0.53	1.00 (0.98, 1.02)	0.17	0.92[d]
Height (age 10–24 years)	Low AN and high BMI	1.00 (1.00, 1.01)	0.15	0.64	1.01 (1.00, 1.01)	0.62	0.01[d]
Height (age 10–24 years)	High AN and high BMI	1.00 (0.99,1.01)	−0.25	0.72	1.00 (0.99, 1.01)	−0.25	0.72
Height (age 10–24 years)	High AN and low BMI	1.00 (1.00, 1.00)	−0.08	0.79	1.00 (1.00, 1.01)	0.13	0.71

Full description of ALSPAC is provided elsewhere.[16–20] Categorical variables were derived from dichotomizing the AN PGS and the BMI PGS. For both the AN and the BMI PGSs, individuals with scores at or greater than the 8th decile point were regarded as the "high PGS group," and those with scores lower were considered the "low PGS group." From the dichotomized AN PGS and BMI PGS, we were able to create a categorical variable with four levels: (1) low AN PGS and low BMI PGS, (2) high AN PGS and low BMI PGS, (3) high AN PGS and high BMI PGS, and (4) low AN PGS and high BMI PGS. The "low AN PGS and low BMI PGS" group was used as the reference category in the analyses. AN, anorexia nervosa; PGS, polygenic score; BMI, body mass index (weight in kilograms/height^2 in meters); FMI, fat mass index (fat mass in kilograms/height^2 in meters); LMI, lean mass index (lean mass in kilograms/height^2 in meters); BMD, bone mineral density (gram/cm^2).

[a]Betas reflect change in outcome compared to the reference category "low AN PGS and low BMI PGS."

[b]Considering that the outcomes were log-transformed we report the percent change in the outcome in the comparison to the reference group ("low AN PGS and BMI PGS") to ease the interpretation of the betas.

[c]Benjamini & Hochberg false discovery rate adjusting for the number of hypotheses tested.[34]

[d]Significant after accounting for multiple testing using the false discovery rate-corrected Q values. Significance was set at $Q < 0.05$.

environmental and genetic factors.[31,37] In a recent study, we reported a negative genetic correlation between body fat percentage and AN, which was significantly more pronounced in women than in men (women SNP-$r_g = -0.44$, SE = 0.04; men SNP-$r_g = -0.26$; SE = 0.04).[37] The negative association between the AN PGS and the BMD trajectory is consistent with the established literature that AN is phenotypically associated with BMD; AN has marked and severe adverse effects on bone metabolism.[3,38] The negative association between the AN PGS and BMD was found only in female participants. The observed sex-specific effects of the AN PGS suggest that a specific set of common genetic variants may be differentially active in women and may increase the liability for AN. To ensure that these associations are not driven by minor sex-specific differences as a consequence of the sampling of the AN GWAS (that mostly included female participants), it is important to collect adequate samples from men with AN and all EDs, allowing for the calculation of ED PGSs specific for men, to confirm our findings on sex-specific genetic effects.

We also demonstrate that developmental changes in body composition are in part driven by the BMI PGS as early as 4 months of age. The pattern of association of the BMI PGS with the growth trajectories did not differ between the sexes. The BMI PGS was significantly associated with FMI and LMI trajectories, whereas the AN PGS was not—another point of divergence between the BMI PGS and the AN PGS. The association of the BMI PGS with FMI and LMI is in part due to shared genomics.[37] In a previous study,[37] we showed a significant genetic correlation between childhood BMI and adiposity (SNP-$r_g = 0.46$) and between childhood BMI and lean mass (SNP-$r_g = 0.41$). Consistent with our findings, increases in body weight are reported to be associated with higher BMD.[39] Whether these associations are due to adaptive changes of the body

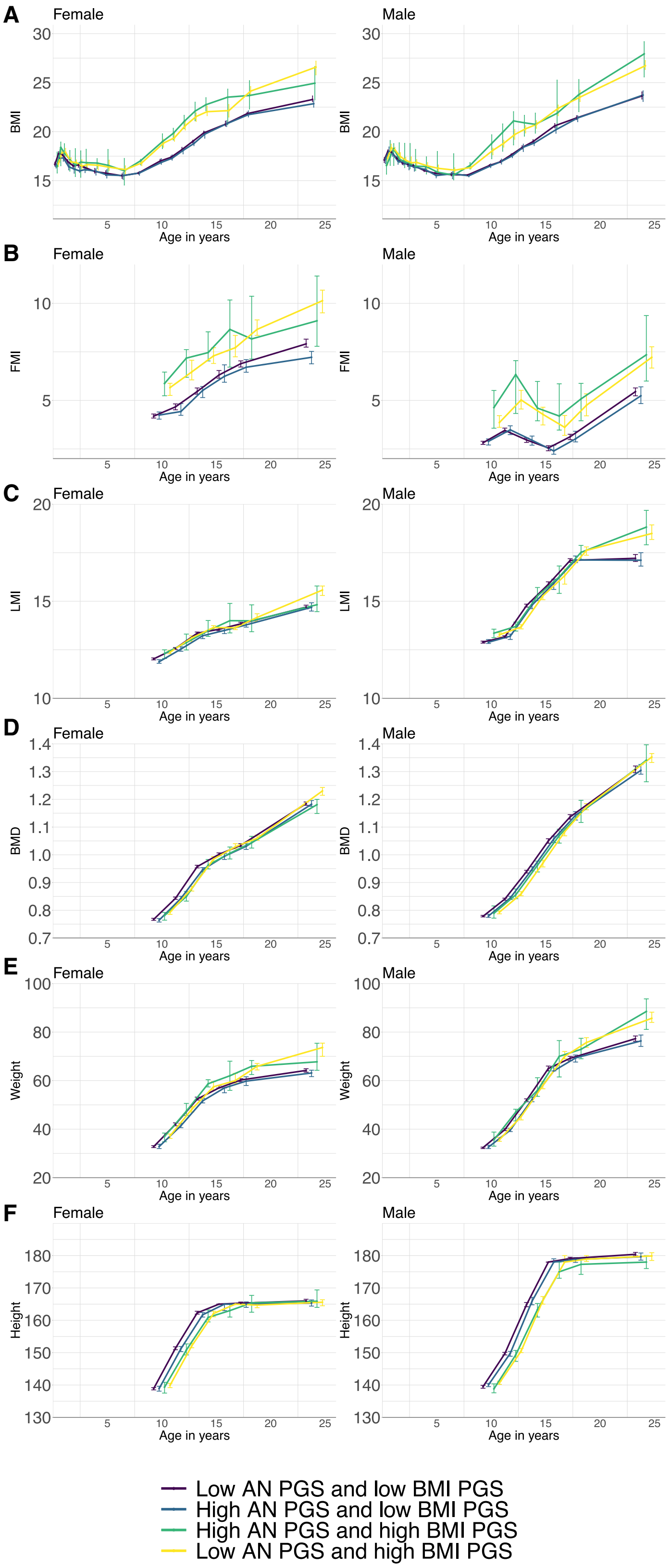

Figure 1. Growth trajectories for the categorial AN and BMI PGS groups

PGS groups were derived by first dichotomizing the AN and the BMI PGSs based on a cut-off value of the 8th decile of the scores. Individuals with a PGS lower than the 8th decile were grouped into a "low PGS" group, and those with a PGS at or higher than 8th decile were grouped into a "high PGS" group. Based on this grouping, we determined four categories: individuals with (1) low PGS for both AN and BMI, (2) high PGS for AN and low PGS for BMI, (3) low PGS for AN and high PGS for BMI, and (4) high PGS for both AN and BMI.

(A) Median (with 95% bootstrapped confidence interval [CI]) BMI (weight in kilograms/height^2 in meters) trajectories across childhood and adolescence.

(B) Median (with 95% bootstrapped CI) FMI (fat mass in kilograms/height^2 in meters) trajectories across childhood and adolescence.

(C) Median (with 95% bootstrapped CI) LMI (lean mass in kilograms/height^2 in meters) trajectories across childhood and adolescence.

(D) Median (with 95% bootstrapped CI) BMD (g/cm^2) trajectories across childhood and adolescence.

(E) Median (with 95% bootstrapped CI) weight (in kilograms) trajectories across childhood and adolescence.

(F) Median (with 95% bootstrapped CI) height (in centimeters) trajectories across childhood and adolescence.

to increased body weight (i.e., higher BMI PGS leads to increases in body weight, which in turn leads to increases in BMD to sustain a higher body weight) or whether this is due to shared genomics is currently unclear.

Furthermore, regression slopes of PGSs and body composition did not differ between males and females, with two exceptions being the association between BMI PGS and BMI trajectory (in which females had a 24% steeper slope) and the FMI trajectory (in which males had a 20% steeper slope). The formula[32,33] used for comparing the slopes depends on standard error estimates and is therefore sensitive to sample size; hence the negative results for the AN PGS might be a reflection of sample size.

We also sought to understand how AN and BMI genetics could co-influence growth across childhood and adolescence. We found that female participants with high AN PGS and low BMI PGS did not differ significantly from a reference group with a low AN PGS and low BMI PGS, although the direction of difference aligned with expectations—individuals with high AN PGS and low BMI PGS have lower growth trajectories compared to the reference group. Interestingly, female participants with high genetic liability for AN and low genetic liability for BMI followed lower growth trajectories than individuals with high PGSs on both traits, which might suggest that the BMI PGS mitigated the effects of the AN PGS. This interpretation is consistent with reports of a negative genetic correlation between AN and BMI.[4,40] Taken together, the findings from the univariate AN PGS analyses and the findings from the extreme-group comparisons suggest that genetic liability to high weight exerts a modulating role in the association between the AN PGS and growth trajectories. However, we cannot exclude

Table 5. Associations of the combined AN and BMI PGS with the body composition measures stratified for biological sex using linear mixed models in ALSPAC: Post-hoc analyses of the BMI trajectory

	BMI female				BMI male			
**PGS comparison group**	**Beta**	**SE**	**Z ratio**	**P[a]**	**Beta**	**SE**	**Z ratio**	**P[a]**
Low AN PGS and low BMI PGS—high AN PGS and low BMI PGS	0.002	0.006	0.270	0.99	−0.001	0.006	−0.153	1
Low AN PGS and low BMI PGS—high AN PGS and high BMI PGS	−0.102	0.014	−7.044	<0.001[b]	−0.097	0.012	−7.775	<0.001[b]
Low AN PGS and low BMI PGS—low AN PGS and high BMI PGS	−0.093	0.006	−14.374	<0.001[b]	−0.071	0.006	−12.310	<0.001[b]
High AN PGS and low BMI PGS—high AN PGS and high BMI PGS	−0.103	0.015	−6.806	<0.001[b]	−0.097	0.013	−7.314	<0.001[b]
High AN PGS and low BMI PGS—low AN PGS and high BMI PGS	−0.095	0.008	−11.690	<0.001[b]	−0.071	0.007	−9.707	<0.001[b]
High AN PGS and high BMI PGS—low AN PGS and high BMI PGS	0.009	0.015	0.567	0.94	0.026	0.013	1.989	0.19

Full description of ALSPAC is provided elsewhere.[16–20] Shown here are the post-hoc comparisons of the PGS groups for only the BMI trajectory (see also Table 4). The full results for the remaining trajectories (FMI, LMI, BMD, weight, and height) can be found in Table S3. Categorical variables were derived from dichotomizing the AN PGS and the BMI PGS. For both the AN and the BMI PGSs, individuals with PGS scores at or greater than the 8th decile point were regarded as the "high PGS group," and those with scores lower were considered the "low PGS group." From the dichotomized AN PGS and BMI PGS, we were able to create a categorical variable with two levels: (1) high AN PGS and high BMI PGS and (2) high AN PGS and low BMI PGS. AN, anorexia nervosa; PGS, polygenic score; BMI, body mass index (weight in kilograms/height^2 in meters); SE, standard error.
[a]Post-hoc comparisons corrected for multiple testing using Tukey's adjustment.
[b]Significant after accounting for multiple testing.

that other unmeasured PGSs (e.g., depression) could also exert influence on these associations.[41]

The observation that AN PGS and BMI PGS co-influence growth trajectories is an interesting finding that encourages exploration of the underlying biological mechanisms. The fact that a PGS of one trait could mitigate the effects of another trait is intriguing and has previously been described in a study investigating stressful life events; a higher well-being PGS buffered against increased depressive symptoms following a spouse's death.[42] Common genomic variants that are associated with AN or BMI are primarily expressed in the central nervous system[4,13,43] suggesting that body mass is behaviorally influenced. This view is also supported by our previous work and that of others, showing that a PGS for a higher BMI is associated with increased food-approach eating behaviors such as emotional eating as well as disordered eating behaviors such as higher propensity to engage in self-reported binge eating.[44–46] Research also indicates that a higher PGS for AN is associated with emotional eating[44] and childhood fussy eating.[47] Taken together, several sources of converging evidence suggest that polygenic risk for AN and/or BMI may impact growth at least in part via eating behaviors.

The biology behind sex-specific effects in AN is not fully understood. From the literature it is clear that AN occurs more often in females than in males (male-to-female ratios of 1:8), and it is likely that some of this sex discrepancy in risk is due to genetics.[48] One possible explanation could be that there is an interaction between common genetic variants of AN (as captured by our AN PGS) on the autosomes and variants on the X chromosomes that differentially impact growth in females compared to males. Another possible explanation for the sex-specific effects observed in this study may be due to increases in ovarian hormones in females during puberty.[49] Klump et al.[49] found that genetic influence on disordered eating increases in females during puberty, whereas no significant difference in genetic influence was observed in males during the same period.[49]

This study has several strengths, including the large sample size and the prospective and repeated collection of objective body composition measures spanning more than 20 years. We included a negative control (i.e., the height trajectory, which was not associated with the AN PGS), adequately controlled for multiple testing, and controlled for potential genetic confounders using ancestry-informative principal components. The use of splines enabled modeling of BMI trajectories more accurately, as growth throughout childhood is not linear. Further, by stratifying on biological sex, we were able to identify sex-related effects that otherwise would have been masked, and censoring on EDs ruled out that growth changes during puberty were a consequence of an ED. We would also like to highlight that the sex differences observed for the AN PGS are likely not due to the sampling of the GWAS of AN that mostly included female participants; the authors of the GWAS of AN, in their sensitivity analysis, found no differences in polygenic architecture between female and male AN cases.[4] However, this does not exclude that the sampling of the AN GWAS could have caused minor sex-specific differences in our PGS analysis.

Findings from this study should be interpreted in the context of some limitations. Participants were recruited from the same geographical region in the southwest of England, and therefore the results may not be generalizable to other populations. However, the homogeneity of this sample lends itself to genetic analyses as bias from population

stratification is low.[50] Considering the longitudinal nature of the study, participants tend to drop out over time, leading to missing data. We maximized available data by using mixed-effects models, which allowed us to use all available data points in deriving the growth trajectories rather than only including complete cases. We acknowledge that any bias as a consequence of missing data in our analyses could have biased our results toward the null.

The effect sizes observed in this study were relatively small, but they are consistent with those previously reported in other PGS studies and aid our understanding of growth trajectories.[15,51] Height and weight data in ALSPAC were obtained from a range of clinical sources, which could have introduced variability in the obtained measures. However, measures of height and weight were highly correlated across different measurement settings (clinic and self-report) (Figure S1). Furthermore, the BMI GWAS that was used in deriving the PGS was conducted mostly in adult participants, which could have biased our analyses. However, for BMI, a previous study found substantial overlap between childhood and adult BMI GWAS loci ($R_g = 0.76$, $p = 1.45 \times 10^{-112}$).[52] Regarding the AN GWAS, it is important to note that both adolescents and children were included in the study; therefore, the AN GWAS should be equipped to pick up genomic variants associated with the disorder in adolescence, which is the typical age of onset.

In conclusion, we show that polygenic risk for AN and BMI has detectable sex-specific effects on growth during the first two decades of life. Especially noteworthy, female participants with high polygenic risk for AN and a low polygenic risk for BMI likely constitute a high-risk group, as they followed lower growth trajectories, which have previously been associated with AN in the ALSPAC sample.[7] This study adds to a growing body of evidence suggesting that risk for AN could emerge during early childhood and that a combination of AN and BMI polygenic risk could aid the early identification of individuals at high risk for AN. These findings encourage further research to understand how AN PGS and BMI PGS co-influence growth during childhood and how BMI PGS can amplify or mitigate the effects of AN PGS.

Data and code availability

Code supporting the current study is available upon request. Data supporting the current study is available from ALSPAC. For more information, visit the ALSPAC study website: http://www.bristol.ac.uk/alspac/researchers/our-data/.

Supplemental information

Supplemental information can be found online at https://doi.org/10.1016/j.ajhg.2022.05.005.

Acknowledgments

We are extremely grateful to all of the families who took part in this study, the midwives for their help in recruiting them, and the whole ALSPAC team, which includes interviewers, computer and laboratory technicians, clerical workers, research scientists, volunteers, managers, receptionists, and nurses. This study represents independent research partly funded by the UK National Institute for Health Research (NIHR) Biomedical Research Centre at South London and Maudsley NHS Foundation Trust and King's College London. High-performance computing facilities were funded with capital equipment grants from the GSTT Charity (TR130505) and Maudsley Charity (980). This work was supported by the UK Medical Research Council and the Medical Research Foundation (MR/R004803/1). The UK Medical Research Council and Wellcome (102215/2/13/2 and 217065/Z/19/Z) and the University of Bristol provide core support for ALSPAC. A comprehensive list of grants is available on the ALSPAC website. This research was specifically funded by the NIHR (CS/01/2008/014) and the NIH (MH087786-01). GWAS data were generated by sample logistics and genotyping facilities at Wellcome Sanger Institute and LabCorp (Laboratory Corporation of America) using support from 23andMe. N.M. and C.M.B. acknowledge funding from the National Institute of Mental Health (R21 MH115397). C.M.B. is supported by NIMH (R01MH120170, R01MH124871, R01M H119084, R01MH118278, R01 MH124871), Brain and Behavior Research Foundation Distinguished Investigator Grant, Swedish Research Council (Vetenskapsrådet, 538-2013-8864), and Lundbeck Foundation (R276-2018-4581). The content is solely the responsibility of the authors and does not necessarily represent the official views of the National Institutes of Health, the UK NHS, the NIHR, or the Department of Health. The funders were not involved in the design or conduct of the study; collection, management, analysis, or interpretation of the data; or preparation, review, or approval of the manuscript.

Author contributions

M.A., C.H., and M.H. analyzed the data. M.A., C.H., and M.H. drafted the manuscript. N.M., C.M.B., R.J.F.L., and G.B. supervised the work. All authors substantially contributed to the conception and interpretation of the work, revised the manuscript for important intellectual content, and approved the final version. All authors agree to be accountable for all aspects of this work.

Declaration of interests

G.B. has received grant funding from and served as a consultant to Eli Lilly, has received honoraria from Illumina, and has served on advisory boards for Otsuka. C.M.B. is a grant recipient from and has served on advisory boards for Takeda/Shire and is a consultant for Idorsia. She receives royalties from Pearson. She is on the Clinical Advisory Board of Equip Health Inc.

Received: December 20, 2021
Accepted: May 9, 2022
Published: June 14, 2022

References

1. Treasure, J., Zipfel, S., Micali, N., Wade, T., Stice, E., Claudino, A., Schmidt, U., Frank, G.K., Bulik, C.M., and Wentz, E. (2015). Anorexia nervosa. Nat. Rev. Dis. Prim. *1*, 15074–15122. https://doi.org/10.1038/nrdp.2015.74.

2. Polito, A., Cuzzolaro, M., Raguzzini, A., Censi, L., and Ferro-Luzzi, A. (1998). Body composition changes in anorexia nervosa. Eur. J. Clin. Nutr. *52*, 655–662. https://doi.org/10.1038/sj.ejcn.1600618.
3. Hübel, C., Yilmaz, Z., Schaumberg, K.E., Breithaupt, L., Hunjan, A., Horne, E., García-González, J., O'Reilly, P.F., Bulik, C.M., and Breen, G. (2019). Body composition in anorexia nervosa: meta-analysis and meta-regression of cross-sectional and longitudinal studies. Int. J. Eat. Disord. *52*, 1205–1223. https://doi.org/10.1002/eat.23158.
4. Watson, H.J., Yilmaz, Z., Thornton, L.M., Hübel, C., Coleman, J.R.I.I., Gaspar, H.A., Bryois, J., Hinney, A., Leppä, V.M., Mattheisen, M., et al. (2019). Genome-wide association study identifies eight risk loci and implicates metabo-psychiatric origins for anorexia nervosa. Nat. Genet. *51*, 1207–1214. https://doi.org/10.1038/s41588-019-0439-2.
5. Stice, E. (2016). Interactive and mediational etiologic models of eating disorder onset: evidence from prospective studies. Annu. Rev. Clin. Psychol. *12*, 359–381. https://doi.org/10.1146/annurev-clinpsy-021815-093317.
6. Tyrka, A.R., Waldron, I., Graber, J.A., and Brooks-Gunn, J. (2002). Prospective predictors of the onset of anorexic and bulimic syndromes. Int. J. Eat. Disord. *32*, 282–290. https://doi.org/10.1002/eat.10094.
7. Yilmaz, Z., Gottfredson, N.C., Zerwas, S.C., Bulik, C.M., and Micali, N. (2019). Developmental premorbid body mass index trajectories of adolescents with eating disorders in a longitudinal population cohort. J. Am. Acad. Child Adolesc. Psychiatry *58*, 191–199. https://doi.org/10.1016/j.jaac.2018.11.008.
8. Simon, G.E., Von Korff, M., Saunders, K., Miglioretti, D.L., Crane, P.K., Van Belle, G., and Kessler, R.C. (2006). Association between obesity and psychiatric disorders in the US adult population. Arch. Gen. Psychiatry *63*, 824–830. https://doi.org/10.1001/archpsyc.63.7.824.
9. González-Muniesa, P., Mártinez-González, M.-A., Hu, F.B., Després, J.-P., Matsuzawa, Y., Loos, R.J.F., Moreno, L.A., Bray, G.A., and Martinez, J.A. (2017). Obes. Nat. Rev. Dis. Prim. *3*, 17034. https://doi.org/10.1038/nrdp.2017.34.
10. Tomiyama, A.J., Carr, D., Granberg, E.M., Major, B., Robinson, E., Sutin, A.R., and Brewis, A. (2018). How and why weight stigma drives the obesity "epidemic" and harms health. BMC Med. *16*, 123. https://doi.org/10.1186/s12916-018-1116-5.
11. Spahlholz, J., Baer, N., König, H.H., Riedel-Heller, S.G., and Luck-Sikorski, C. (2016). Obesity and discrimination - a systematic review and meta-analysis of observational studies. Obes. Rev. *17*, 43–55. https://doi.org/10.1111/obr.12343.
12. Daly, M., Sutin, A.R., and Robinson, E. (2019). Perceived weight discrimination mediates the prospective association between obesity and physiological dysregulation: evidence from a population-based cohort. Psychol. Sci. *30*, 1030–1039. https://doi.org/10.1177/0956797619849440.
13. Locke, A.E., Kahali, B., Berndt, S.I., Justice, A.E., Pers, T.H., Day, F.R., Powell, C., Vedantam, S., Buchkovich, M.L., Yang, J., et al. (2015). Genetic studies of body mass index yield new insights for obesity biology. Nature *518*, 197–206. https://doi.org/10.1038/nature14177.
14. Yengo, L., Sidorenko, J., Kemper, K.E., Zheng, Z., Wood, A.R., Weedon, M.N., Frayling, T.M., Hirschhorn, J., Yang, J., and Visscher, P.M. (2018). Meta-analysis of genome-wide association studies for height and body mass index in ∼700000 individuals of European ancestry. Hum. Mol. Genet. *27*, 3641–3649. https://doi.org/10.1093/hmg/ddy271.
15. Khera, A.V., Chaffin, M., Wade, K.H., Zahid, S., Brancale, J., Xia, R., Distefano, M., Senol-Cosar, O., Haas, M.E., Bick, A., et al. (2019). Polygenic prediction of weight and obesity trajectories from birth to adulthood. Cell *177*, 587–596.e9. https://doi.org/10.1016/j.cell.2019.03.028.
16. Fraser, A., Macdonald-wallis, C., Tilling, K., Boyd, A., Golding, J., Davey smith, G., Henderson, J., Macleod, J., Molloy, L., Ness, A., et al. (2013). Cohort profile: the avon longitudinal study of parents and children: ALSPAC mothers cohort. Int. J. Epidemiol. *42*, 97–110. https://doi.org/10.1093/ije/dys066.
17. Boyd, A., Golding, J., Macleod, J., Lawlor, D.A., Fraser, A., Henderson, J., Molloy, L., Ness, A., Ring, S., and Davey Smith, G. (2013). Cohort profile: the 'Children of the 90s'-The index offspring of the avon longitudinal study of parents and children. Int. J. Epidemiol. *42*, 111–127. https://doi.org/10.1093/ije/dys064.
18. Golding, J., Pembrey, M., Jones, R.; and The Alspac Study Team (2001). ALSPAC-the avon longitudinal study of parents and children. Paediatr. Perinat. Epidemiol. *15*, 74–87. https://doi.org/10.1046/j.1365-3016.2001.00325.x.
19. Golding, J. (2004). The avon longitudinal study of parents and children (ALSPAC)–study design and collaborative opportunities. Eur. J. Endocrinol., U119–U123. https://doi.org/10.1530/eje.0.151u119.
20. Northstone, K., Lewcock, M., Groom, A., Boyd, A., Macleod, J., Timpson, N., and Wells, N. (2019). The Avon Longitudinal Study of Parents and Children (ALSPAC): an update on the enrolled sample of index children in 2019. Wellcome Open Res. *4*, 51. https://doi.org/10.12688/wellcomeopenres.15132.1.
21. Harris, P.A., Taylor, R., Thielke, R., Payne, J., Gonzalez, N., and Conde, J.G. (2009). Research electronic data capture (REDCap)-A metadata-driven methodology and workflow process for providing translational research informatics support. J. Biomed. Inform. *42*, 377–381. https://doi.org/10.1016/j.jbi.2008.08.010.
22. Harris, P.A., Taylor, R., Minor, B.L., Elliott, V., Fernandez, M., O'Neal, L., McLeod, L., Delacqua, G., Delacqua, F., Kirby, J., and Duda, S.N. (2019). The REDCap consortium: Building an international community of software platform partners. J. Biomed. Inform. *95*, 103208. https://doi.org/10.1016/j.jbi.2019.103208.
23. Micali, N., De Stavola, B., Ploubidis, G., Simonoff, E., Treasure, J., and Field, A.E. (2015). Adolescent eating disorder behaviours and cognitions: Gender-specific effects of child, maternal and family risk factors. Br. J. Psychiatry *207*, 320–327. https://doi.org/10.1192/bjp.bp.114.152371.
24. Micali, N., Solmi, F., Horton, N.J., Crosby, R.D., Eddy, K.T., Calzo, J.P., Sonneville, K.R., Swanson, S.A., and Field, A.E. (2015). Adolescent eating disorders predict psychiatric, high-risk behaviors and weight outcomes in young adulthood. J. Am. Acad. Child Adolesc. Psychiatry *54*, 652–659.e1. https://doi.org/10.1016/j.jaac.2015.05.009.
25. Hübel, C., Abdulkadir, M., Herle, M., Loos, R.J.F., Breen, G., Bulik, C.M., and Micali, N. (2021). One size does not fit all. Genomics differentiates among anorexia nervosa, bulimia nervosa, and binge-eating disorder. Int. J. Eat. Disord. *54*, 785–793. https://doi.org/10.1002/eat.23481.

26. Warrington, N.M., Howe, L.D., Wu, Y.Y., Timpson, N.J., Tilling, K., Pennell, C.E., Newnham, J., Davey-Smith, G., Palmer, L.J., Beilin, L.J., et al. (2013). Association of a body mass index genetic risk score with growth throughout childhood and adolescence. PLoS One *8*, e79547. https://doi.org/10.1371/journal.pone.0079547.
27. Ge, T., Chen, C.Y., Ni, Y., Feng, Y.C.A., and Smoller, J.W. (2019). Polygenic prediction via Bayesian regression and continuous shrinkage priors. Nat. Commun. *10*, 1776–1810. https://doi.org/10.1038/s41467-019-09718-5.
28. Kahn, R.S., Sommer, I.E., Murray, R.M., Meyer-Lindenberg, A., Weinberger, D.R., Cannon, T.D., O'Donovan, M., Correll, C.U., Kane, J.M., van Os, J., and Insel, T.R. (2015). Schizophrenia. Nat. Rev. Dis. Prim. *1*, 15067. https://doi.org/10.1038/nrdp.2015.67.
29. Bates, D., Mächler, M., Bolker, B.M., and Walker, S.C. (2015). Fitting Linear Mixed-Effects Models Using Lme4. Journal of Statistical Software *67*, 1–48. https://doi.org/10.18637/jss.v067.i01.
30. Hübel, C., Gaspar, H.A., Coleman, J.R.I., Hanscombe, K.B., Purves, K., Prokopenko, I., Graff, M., Ngwa, J.S., Workalemahu, T., O'Reilly, P.F., et al. (2019). Genetic correlations of psychiatric traits with body composition and glycemic traits are sex- and age-dependent. Nat. Commun. *10*, 5765. https://doi.org/10.1038/s41467-019-13544-0.
31. Zillikens, M.C., Yazdanpanah, M., Pardo, L.M., Rivadeneira, F., Aulchenko, Y.S., Oostra, B.A., Uitterlinden, A.G., Pols, H.A.P., and Van Duijn, C.M. (2008). Sex-specific genetic effects influence variation in body composition. Diabetologia *51*, 2233–2241. https://doi.org/10.1007/s00125-008-1163-0.
32. Clogg, C.C., Petkova, E., and Haritou, A. (1995). Statistical methods for comparing regression coefficients between models. Am. J. Sociol. *100*, 1261–1293. https://doi.org/10.1086/230638.
33. Paternoster, R., Brame, R., Mazerolle, P., and Piquero, A. (1998). Using the correct statistical test for the equality of regression coefficients. Criminology *36*, 859–866. https://doi.org/10.1111/j.1745-9125.1998.tb01268.x.
34. Benjamini, Y., and Hochberg, Y. (1995). Controlling the false discovery rate : a Practical and powerful approach to multiple testing. J. R. Stat. Soc. Ser. B *57*, 289–300. https://doi.org/10.1111/j.2517-6161.1995.tb02031.x.
35. Herle, M., Micali, N., Abdulkadir, M., Loos, R., Bryant-Waugh, R., Hübel, C., Bulik, C.M., and De Stavola, B.L. (2020). Identifying typical trajectories in longitudinal data: modelling strategies and interpretations. Eur. J. Epidemiol. *35*, 205–222. https://doi.org/10.1007/s10654-020-00615-6.
36. Wells, J.C.K. (2007). Sexual dimorphism of body composition. Best Pract. Res. Clin. Endocrinol. Metab. *21*, 415–430. https://doi.org/10.1016/j.beem.2007.04.007.
37. Hübel, C., Gaspar, H.A., Coleman, J.R.I.I., Finucane, H., Purves, K.L., Hanscombe, K.B., Prokopenko, I., Graff, M., Ngwa, J.S., Workalemahu, T., et al. (2019). Genomics of body fat percentage may contribute to sex bias in anorexia nervosa. Am. J. Med. Genet. Part B Neuropsychiatr. Genet. *180*, 428–438. https://doi.org/10.1002/ajmg.b.32709.
38. Bulik, C.M., Carroll, I.M., and Mehler, P. (2021). Endocrinology & Metabolism Reframing anorexia nervosa as a metabo-psychiatric disorder. Trends Endocrinol. Metab. *xx*, 1–10.
39. Maïmoun, L., Garnero, P., Mura, T., Nocca, D., Lefebvre, P., Philibert, P., Seneque, M., Gaspari, L., Vauchot, F., Courtet, P., et al. (2020). Specific effects of anorexia nervosa and obesity on bone mineral density and bone turnover in young women. J. Clin. Endocrinol. Metab. *105*, e1536–e1548. https://doi.org/10.1210/clinem/dgz259.
40. Duncan, L., Yilmaz, Z., Gaspar, H., Walters, R., Goldstein, J., Anttila, V., Bulik-Sullivan, B., Ripke, S., Thornton, L., Hinney, A., et al. (2017). Significant locus and metabolic genetic correlations revealed in genome-wide association study of anorexia nervosa. Am. J. Psychiatry *174*, 850–858. https://doi.org/10.1176/appi.ajp.2017.16121402.
41. Pine, D.S., Goldstein, R.B., Wolk, S., and Weissman, M.M. (2001). The association between childhood depression and adulthood body mass index. Pediatrics *107*, 1049–1056. https://doi.org/10.1542/peds.107.5.1049.
42. Domingue, B.W., Liu, H., Okbay, A., and Belsky, D.W. (2017). Genetic Heterogeneity in depressive symptoms following the death of a spouse: polygenic score analysis of the U.S. health and retirement study. Am. J. Psychiatry *174*, 963–970. https://doi.org/10.1176/appi.ajp.2017.16111209.
43. Goodarzi, M.O. (2018). Genetics of obesity: what genetic association studies have taught us about the biology of obesity and its complications. Lancet Diabetes Endocrinol. *6*, 223–236. https://doi.org/10.1016/s2213-8587(17)30200-0.
44. Yilmaz, Z., Schaumberg, K., Halvorsen, M., Goodman, E.L., Brosof, L.C., Crowley, J.J., Mathews, C.A., Mattheisen, M., Breen, G., Bulik, C.M., et al. (2022). Predicting eating disorder and anxiety symptoms using disorder-specific and transdiagnostic polygenic scores for anorexia nervosa and obsessive-compulsive disorder. Psychol. Med., 1–15. https://doi.org/10.1017/s0033291721005079.
45. Herle, M., Smith, A.D., Kininmonth, A., and Llewellyn, C. (2020). The role of eating behaviours in genetic susceptibility to obesity. Curr. Obes. Rep. *9*, 512–521. https://doi.org/10.1007/s13679-020-00402-0.
46. Abdulkadir, M., Herle, M., De Stavola, B.L., Hübel, C., Santos Ferreira, D.L., Loos, R.J.F., Bryant-Waugh, R., Bulik, C.M., and Micali, N. (2020). Polygenic score for body mass index is associated with disordered eating in a general population cohort. J. Clin. Med. *9*, 1187. https://doi.org/10.3390/jcm9041187.
47. Herle, M., Abdulkadir, M., Hübel, C., Ferreira, D.S., Bryant-Waugh, R., Loos, R.J.F., Bulik, C.M., De Stavola, B., and Micali, N. (2021). The genomics of childhood eating behaviours. Nat. Hum. Behav. *5*, 625–630. https://doi.org/10.1038/s41562-020-01019-y.
48. Steinhausen, H.-C., and Jensen, C.M. (2015). Time trends in lifetime incidence rates of first-time diagnosed anorexia nervosa and bulimia nervosa across 16 years in a danish nationwide psychiatric registry study. Int. J. Eat. Disord. *48*, 845–850. https://doi.org/10.1002/eat.22402.
49. Klump, K.L., Culbert, K.M., Slane, J.D., Burt, S.A., Sisk, C.L., and Nigg, J.T. (2012). The effects of puberty on genetic risk for disordered eating: evidence for a sex difference. Psychol. Med. *42*, 627–637. https://doi.org/10.1017/s0033291711001541.
50. Hellwege, J.N., Keaton, J.M., Giri, A., Gao, X., Velez Edwards, D.R., and Edwards, T.L. (2017). Population stratification in genetic association studies. Curr. Protoc. Hum. Genet. *95*, 1–22. https://doi.org/10.1002/cphg.48.1-1.22.23.

51. Stergiakouli, E., Martin, J., Hamshere, M.L., Heron, J., St Pourcain, B., Timpson, N.J., Thapar, A., and Davey Smith, G. (2016). Association between polygenic risk scores for attention-deficit hyperactivity disorder and educational and cognitive outcomes in the general population. Int. J. Epidemiol. *09*, dyw216. https://doi.org/10.1093/ije/dyw216.

52. Vogelezang, S., Bradfield, J.P., Ahluwalia, T.S., Curtin, J.A., Lakka, T.A., Grarup, N., Scholz, M., van der Most, P.J., Monnereau, C., Stergiakouli, E., et al. (2020). Novel loci for childhood body mass index and shared heritability with adult cardiometabolic traits. PLoS Genet *16*, e1008718. https://doi.org/10.1371/journal.pgen.1008718.

The American Journal of Human Genetics, Volume *109*

Supplemental information

The impact of anorexia nervosa and BMI polygenic risk on childhood growth: A 20-year longitudinal population-based study

Mohamed Abdulkadir, Christopher Hübel, Moritz Herle, Ruth J.F. Loos, Gerome Breen, Cynthia M. Bulik, and Nadia Micali

Supplemental information

Supplemental figures

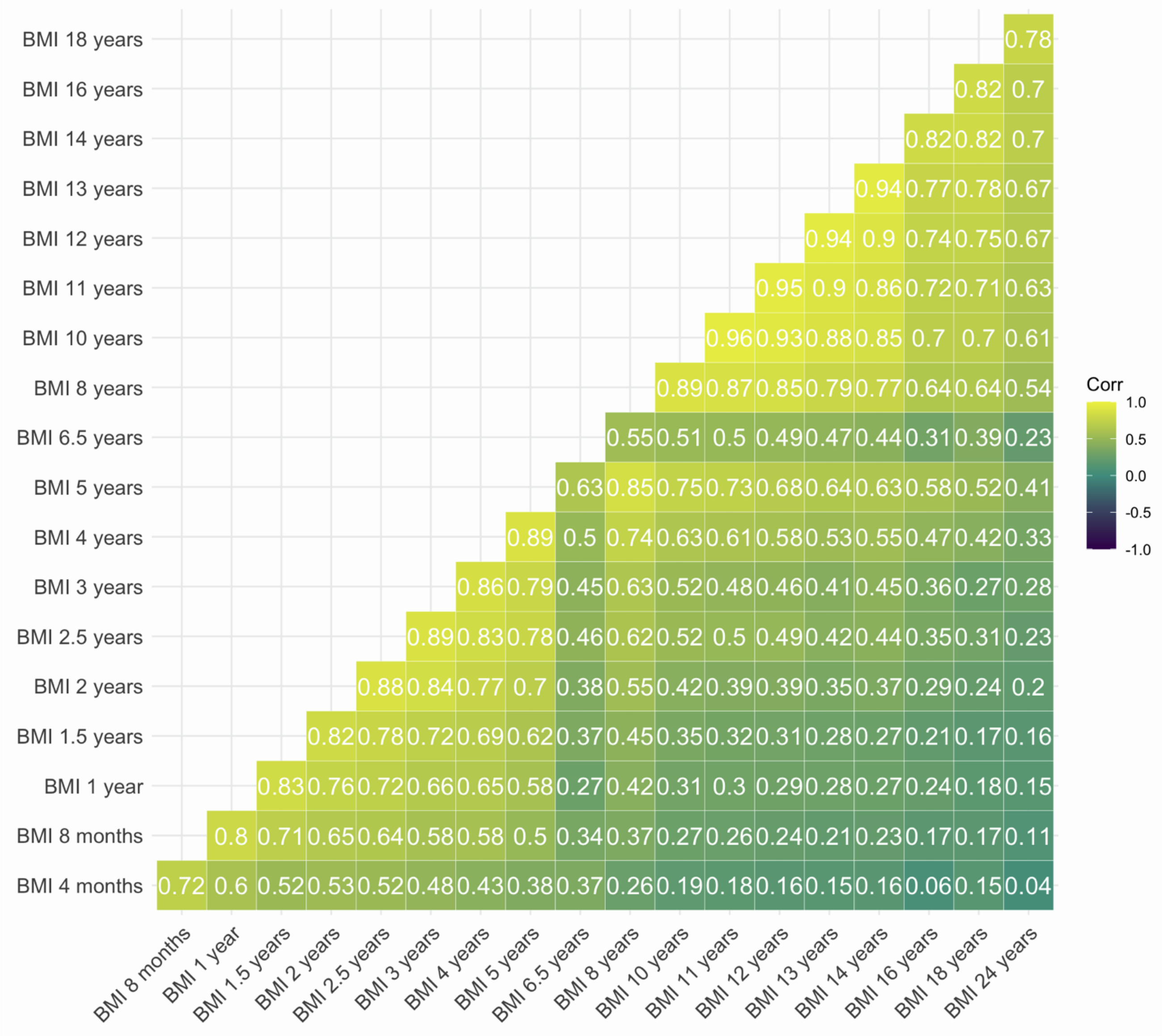

Figure S1: Correlation matrix of body mass index (BMI; weight in kilograms/height^2 in meters) across childhood and adolescence in the Avon Longitudinal Study of Parents and Children (ALSPAC). Numerous measurements on weight and height were collected from different sources (i.e., routine clinic visits, information collected from midwives, linkage to child health records) between birth and age 24 years. Information on weight was collected at research clinic visits annually up to the age 14 years and further clinic measurements at the ages 16, 18, and 24 years using the Tanita Body Fat Analyzer (Tanita TBFUK Ltd.) to the nearest 50 grams. During the same clinic visits, height (standing) was measured to the nearest millimeter with shoes and socks removed using a Holtain stadiometer (Holtain Ltd, Crymych, Pembs, UK). Information on child and adolescent BMI (weight in kilograms / height squared in meters) was derived using weight and height measurement obtained during clinic visits.

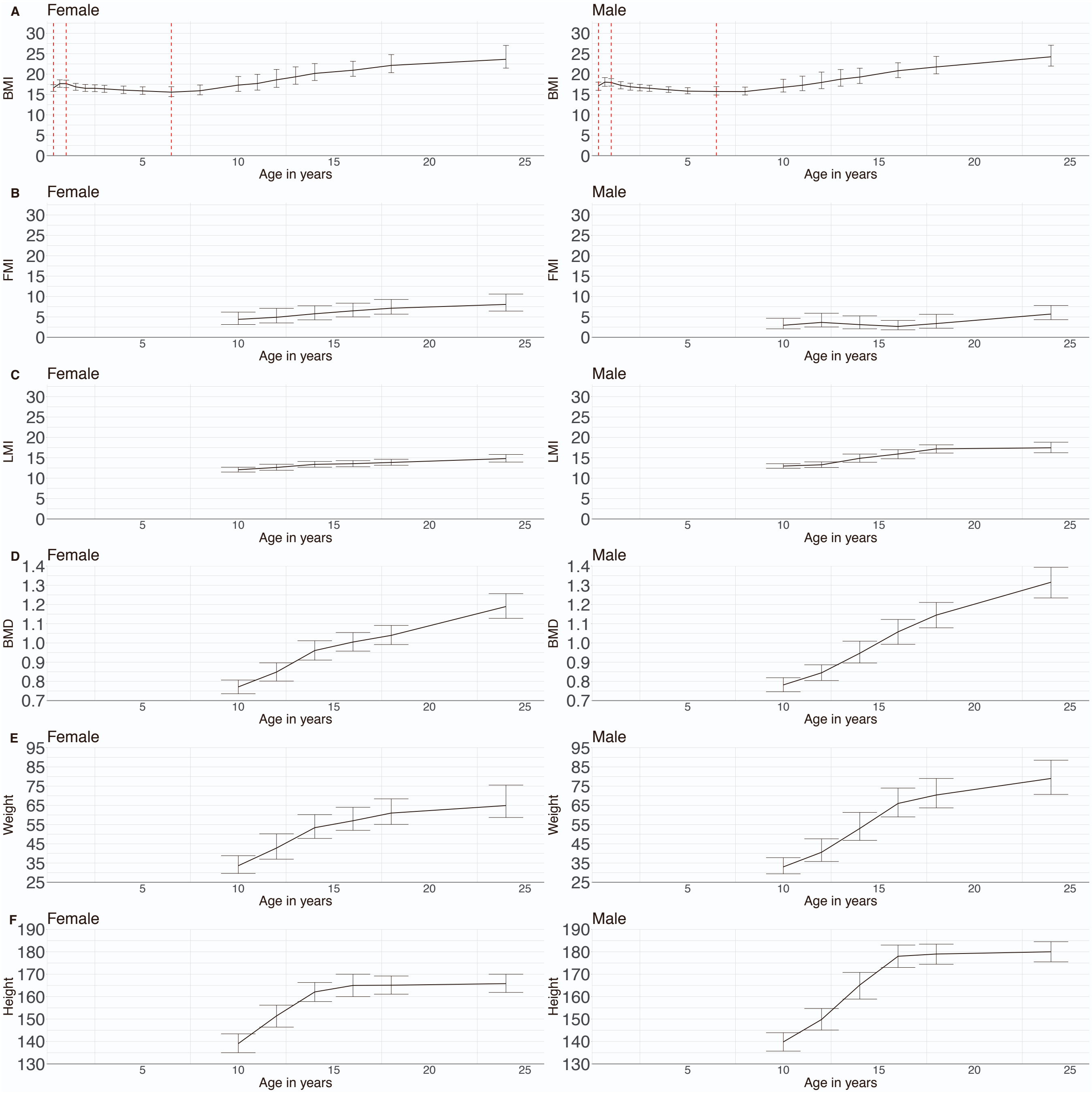

Figure S2: A. Median of body mass index (BMI; weight in kilograms/height^2 in meters) trajectories across childhood and adolescence. The BMI trajectory was derived using spline modeling. Prior to deriving the trajectory. For the spline modeling three spline points (knots shown in red) were placed: knot 1, between age 4 months and 1 year; knot 2, between age 1 year and 6.5 years; and knot 3, between age 6.5 year and 24 years. Intercept reflect BMI value at age 4 months. **B-F** Median fat mass index (FMI; fat mass in kilograms/height^2 in meters), lean mass index (LMI; lean mass in kilograms/height^2 in meters, bone mineral density (BMD; in gram/cm^2), weigh, and height trajectories across childhood and adolescents, respectively. FMI, LMI, BMD, weight, and height trajectory parameters were derived using mixed effects models [7]. Error bars represent the interquartile range.

Supplemental tables

Table S1: Descriptive of the body composition measures of the Avon Longitudinal Study of Parents and Children (ALSPAC)[a]

		Female		Male	
**Body composition measure**	**Age (months)**	**N**	**Median (IQR)**	**N**	**Median (IQR)**
**BMI** [b]	4	337	16.61 (15.76, 17.41)	363	17.12 (16.02, 18.02)
	8	438	17.67 (16.76, 18.6)	491	18.04 (17.01, 19.14)
	12	423	17.67 (16.69, 18.52)	467	17.94 (17.05, 18.89)
	18	405	16.87 (16.03, 17.73)	440	17.25 (16.38, 18.15)
	24	370	16.52 (15.75, 17.47)	411	16.89 (16.06, 17.77)
	30	384	16.53 (15.70, 17.37)	422	16.67 (15.89, 17.48)
	36	385	16.38 (15.53, 17.25)	417	16.53 (15.77, 17.26)
	48	372	16.08 (15.23, 17.06)	419	16.15 (15.52, 16.9)
	60	367	15.88 (15.03, 16.87)	403	15.82 (15.17, 16.67)
	78	544	15.57 (14.49, 16.91)	534	15.73 (14.86, 16.93)
	96	3,130	15.92 (14.92, 17.32)	3,224	15.72 (14.88, 16.82)
	120	3,072	17.31 (15.77, 19.41)	3,026	16.77 (15.61, 18.73)
	132	2,977	17.7 (16.07, 19.94)	2,923	17.26 (15.93, 19.50)
	144	2,902	18.6 (16.74, 21.14)	2,797	17.96 (16.43, 20.50)
	156	2,742	19.34 (17.52, 21.78)	2,630	18.76 (17.04, 21.12)
	168	2,433	20.17 (18.43, 22.58)	2,394	19.28 (17.71, 21.45)
	192	1,496	20.95 (19.47, 23.14)	1,289	20.83 (19.14, 22.78)
	216	1,986	22.14 (20.35, 24.78)	1,681	21.76 (20.08, 24.34)
	288	1,671	23.63 (21.47, 27.04)	1,131	24.25 (21.97, 27.09)
**FMI** [c]	120	2,932	4.40 (3.14, 6.18)	2,880	2.96 (2.08, 4.67)
	144	2,862	4.91 (3.52, 7.11)	2,750	3.65 (2.53, 5.90)
	168	2,406	5.77 (4.28, 7.74)	2,353	3.11 (2.09, 5.26)
	192	1,266	6.50 (5.00, 8.36)	1,066	2.67 (1.83, 4.14)
	216	1,895	7.13 (5.68, 9.28)	1,624	3.37 (2.22, 5.64)
	288	1,618	8.07 (6.43, 10.61)	1,100	5.70 (4.32, 7.80)
**LMI** [c]	120	2,932	12.07 (11.5, 12.69)	2,880	12.98 (12.43, 13.55)
	144	2,862	12.64 (11.95, 13.42)	2,750	13.28 (12.63, 14.00)
	168	2,406	13.39 (12.70, 14.10)	2,353	14.87 (13.90, 15.92)
	192	1,266	13.56 (12.81, 14.3)	1,066	15.93 (14.76, 16.98)
	216	1,895	13.86 (13.17, 14.64)	1,624	17.19 (16.16, 18.18)
	288	1,618	14.8 (13.95, 15.82)	1,100	17.45 (16.25, 18.83)

**BMD** [c]	120	2,965	0.77 (0.74, 0.81)	2,900	0.78 (0.75, 0.82)
	144	2,865	0.85 (0.80, 0.90)	2,756	0.84 (0.80, 0.89)
	168	2,406	0.96 (0.91, 1.01)	2,354	0.95 (0.90, 1.01)
	192	2,050	1.00 (0.96, 1.05)	1,952	1.06 (0.99, 1.12)
	216	1,902	1.04 (0.99, 1.09)	1,634	1.14 (1.08, 1.21)
	288	1,619	1.19 (1.13, 1.26)	1,101	1.32 (1.23, 1.39)
**Weight (in kilograms)** [d]	120	3,105	33.60 (29.6, 38.8)	3,045	33 (29.40, 37.80)
	144	2,904	42.80 (37.00, 50.20)	2,801	40.60 (35.80, 47.60)
	168	2,433	53.40 (47.80, 60.20)	2,394	53.00 (46.80, 61.35)
	192	1,599	57.00 (52.00, 64.00)	1,330	66.00 (59.00, 74.00)
	216	1,986	61.00 (55.10, 68.40)	1,683	70.40 (63.70, 79.00)
	288	1,671	64.90 (58.70, 75.55)	1,131	79.00 (70.70, 88.50)
**Height (in centimeters)** [d]	120	3,074	139.05 (135.0, 143.4)	3,027	139.8 (135.7, 143.9)
	144	2,904	151.4 (146.4, 156.2)	2,797	149.8 (145.1, 154.7)
	168	2,438	162.1 (157.8, 166.3)	2,394	165.2 (158.9, 170.8)
	192	1,666	165.0 (160.0, 170.0)	1,361	178.0 (173.0, 183.0)
	216	1,988	165.1 (161.1, 169.2)	1,683	179.0 (174.5, 183.4)
	288	1,672	165.8 (161.9, 170.0)	1,131	180.0 (175.5, 184.5)

IQR = interquartile range; BMI, body mass index (weight in kilograms/height^2 in meters); FMI, fat mass index (fat mass in kilograms/height^2 in meters); LMI, lean mass index (lean mass in kilograms/height^2 in meters); BMD, bone mineral density.

[a]Full description of the Avon Longitudinal Study of Parents and Children (ALSPAC) is described elsewhere; [2–6].

[b]BMI was calculated using objectively measured weight and height that were collected from different sources (i.e., routine clinic visits, information collected from midwives, linkage to child health records) between birth and age 24 years. Information on weight was collected at research clinic visit annually up to the age 14 years and further clinic measurements at the ages 16, 18, and 24 years using the Tanita Body Fat Analyzer (Tanita TBFUK Ltd.) to the nearest 50 grams. During the same clinic visits, height (standing) was measured to the nearest millimeter with shoes and socks removed using a Holtain stadiometer (Holtain Ltd, Crymych, Pembs, UK).

[c]Fat mass, lean mass, and bone mineral density (BMD) were derived using a Lunar Prodigy dual emission x ray absorptiometry (DEXA) scanner (GE Medical Systems Lunar, Madison, WI, USA). Fat mass index (FMI) and lean mass index (LMI) were calculated by dividing each measure (in kilograms) by height^2 (in meters). The bone mineral density (BMD) is calculated for the whole body excluding the head values.

[d]For uniformity of the analyses, we restricted analyses of weight and height to measurements taken at the same ages FMI, LMI, and BMD were measured.

Table S2: Sex comparisons of the associations of the anorexia nervosa polygenic score with body composition in the Avon Longitudinal Study of Parents and Children (ALSPAC) [a]

Trajectory	Parameter	Beta female [b]	Beta on the log scale female [b]	Standard error female	Beta male [b]	Beta Male on the log scale [b]	Standard error male	% difference in slope females compared to males	Z	P
BMI	Slope age 4 months - 1 year	1.0000	7.48E-07	2.42E-06	1.0000	1.01E-06	2.20E-06	-35.03	-0.08	0.94
BMI	Slope age 1 year - 6.5 years	1.0000	1.85E-05	1.68E-05	1.0000	-5.87E-06	1.62E-05	131.73	1.04	0.29
BMI	Slope age 6.5 years - 24 years	1.0000	-4.00E-05	1.22E-05	1.0000	-2.31E-05	1.08E-05	42.25	-1.03	0.30
FMI	Slope age 10 years - 24 years	0.9919	-8.09E-03	7.55E-03	0.9942	-5.79E-03	9.54E-03	28.43	-0.19	0.85
LMI	Slope age 10 years - 24 years	0.9973)	-2.69E-03	1.49E-03	0.9993	-6.51E-04	1.68E-03	75.80	-0.91	0.36
BMD	Slope age 10 years - 24 years	0.9960	-4.02E-03	1.59E-03	0.9986	-1.44E-03	1.84E-03	64.18	-1.06	0.29
Weight	Slope age 10 years - 24 years	0.9937	-6.30E-03	3.62E-03	0.9956	-4.38E-03	4.05E-03	30.48	-0.35	0.73
Height	Slope age 10 years - 24 years	0.9990)	-1.02E-03	8.25E-04	0.9991	-9.10E-04	1.07E-03	10.78	-0.08	0.94

[a] Full description of the Avon Longitudinal Study of Parents and Children is described elsewhere; [2–6].

[b] Comparison of the betas were done on the log scale as the outcomes were log transformed. Reported here are the non-log transformed and the log transformed betas.

Table S3: Post-hoc power analysis of the anorexia nervosa and body mass index polygenic score.

	AN PGS			BMI PGS	
Trajectory	N	Cov12	Power	Cov12	Power
BMI	3072	-0.32	1	0.95	1
FMI	2932	-0.33	1	0.90	1
LMI	2932	-0.12	1	0.54	1
BMD	2965	0.06	0.97	0.39	1
Weight	3105	-0.23	1	0.82	1
Height	3074	-0.11	1	-0.17	1

AN = anorexia nervosa; PGS = polygenic score ; BMI = body mass index; N = sample size target sample (Avon Longitudinal Study of Parents and Children); Cov12 = Covariance between genetic effect sizes in the two samples; Power = Power of the Chi-squared test of association between polygenic score and target trait.

Table S4: Sex comparisons of the associations of the body mass index polygenic score with body composition in the Avon Longitudinal Study of Parents and Children (ALSPAC) [a]

Trajectory	Parameter	Beta female [b]	Beta on the log scale female [b]	Standard error female	Beta male [b]	Beta on the log scale male [b]	Standard error male	% difference in slope females compared to males	Z	P
BMI	Slope age 4 months - 1 year	1.0000	3.92E-05	2.28E-06	1.0000	2.99E-05	2.09E-06	23.72	2.99	0.003
BMI	Slope age 1 year - 6.5 years	1.0002	1.83E-04	1.68E-05	1.0002	1.93E-04	1.60E-05	-5.46	-0.45	0.65
BMI	Slope age 6.5 years - 24 years	1.0002	1.80E-04	1.22E-05	1.0002	1.57E-04	1.07E-05	12.78	1.42	0.15
FMI	Slope age 10 years - 24 years	1.1673	1.55E-01	6.90E-03	1.2040	1.86E-01	8.69E-03	-20.00	-2.79	0.005
LMI	Slope age 10 years - 24 years	1.0211	2.09E-02	1.40E-03	1.0185	1.83E-02	1.62E-03	12.44	1.19	0.23
BMD	Slope age 10 years - 24 years	1.0164	1.63E-02	1.54E-03	1.0142	1.41E-02	1.78E-03	13.50	0.9	0.37
Weight	Slope age 10 years - 24 years	1.0642	6.22E-02	3.37E-03	1.0584	5.68E-02	3.79E-03	8.68	1.08	0.28
Height	Slope age 10 years - 24 years	1.0016	1.62E-03	8.35E-04	1.0023	2.35E-03	1.06E-03	-45.06	-0.53	0.59

[a] Full description of the Avon Longitudinal Study of Parents and Children is described elsewhere; [2–6].

[b] Comparison of the betas were done on the log scale as the outcomes were log transformed. Reported here are the non-log transformed and the log transformed betas.

Table S5: Sample size of the combined anorexia nervosa and body mass index polygenic scores groups in the Avon Longitudinal Study of Parents and Children[a]

	**Female**	**Male**
**PGS category** [b]	**N**	**N**
Low AN / high BMI	778	702
High AN / high BMI	137	114
High AN / low BMI	765	715
Low AN / low BMI	2,749	2,694

[a] Full description of the Avon Longitudinal Study of Parents and Children is described elsewhere; [2–6].

[b] Categorical variable derived from dichotomizing the AN-PGS and the BMI-PGS. For both the AN and the BMI PGS, individuals with scores at or greater than the 8th decile point were regarded as the "high PGS group" while those with scores lower were considered "low PGS group". From the dichotomized AN-PGS and BMI-PGS we were able to create a categorical variable with four levels (1) low AN-PGS / low BMI-PGS (2) high AN-PGS / low BMI-PGS (3) high AN-PGS / high BMI-PGS (4) low AN-PGS / high BMI-PGS. The "low AN-PGS/low BMI-PGS" group was used as the reference category in the extreme analyses.

Table S6: Associations of the combined anorexia nervosa and body mass index polygenic score with the body composition measures stratified for biological sex using linear mixed models in the Avon Longitudinal Study of Parents and Children: Post-hoc analyses of the BMI trajectory [a b]

		**Female**				**Male**			
**Trajectory**	**Comparison** [b]	**Beta**	**SE**	**Z ratio**	**P** [c]	**Beta**	**SE**	**Z ratio**	**P** [c]
**BMI**	Low AN PGS and low BMI PGS - High AN PGS and low BMI PGS	0.002	0.006	0.270	0.99	-0.001	0.006	-0.153	1
	Low AN PGS and low BMI PGS - High AN PGS and high BMI PGS	-0.102	0.014	-7.044	**<0.001***	-0.098	0.013	-7.775	**<0.001***
	Low AN PGS and low BMI PGS - Low AN PGS and high BMI PGS	-0.093	0.006	-14.375	**<0.001***	-0.071	0.006	-12.308	**<0.001***
	High AN PGS and low BMI PGS - High AN PGS and high BMI PGS	-0.104	0.015	-6.807	**<0.001***	-0.097	0.013	-7.315	**<0.001***
	High AN PGS and low BMI PGS - Low AN PGS and high BMI PGS	-0.095	0.008	-11.690	**<0.001***	-0.071	0.007	-9.708	**<0.001***
	High AN PGS and high BMI PGS - Low AN PGS and high BMI PGS	0.009	0.015	0.567	0.94	0.026	0.013	1.990	0.19
**FMI**	Low AN PGS and low BMI PGS - High AN PGS and low BMI PGS	0.013	0.018	0.720	0.89	-0.015	0.025	-0.613	0.93
	Low AN PGS and low BMI PGS - High AN PGS and high BMI PGS	-0.274	0.042	-6.583	**<0.001***	-0.390	0.053	-7.304	**<0.001***
	Low AN PGS and low BMI PGS - Low AN PGS and high BMI PGS	-0.245	0.019	-13.125	**<0.001***	-0.323	0.025	-13.135	**<0.001***
	High AN PGS and low BMI PGS - High AN PGS and high BMI PGS	-0.287	0.044	-6.563	**<0.001***	-0.375	0.056	-6.675	**<0.001***
	High AN PGS and low BMI PGS - Low AN PGS and high BMI PGS	-0.258	0.023	-11.100	**<0.001***	-0.308	0.031	-10.016	**<0.001***
	High AN PGS and high BMI PGS - Low AN PGS and high BMI PGS	0.029	0.044	0.654	0.91	0.067	0.056	1.194	0.63
**LMI**	Low AN PGS and low BMI PGS - High AN PGS and low BMI PGS	0.006	0.003	1.605	0.38	0.000	0.003	-0.008	**1**
	Low AN PGS and low BMI PGS - High AN PGS and high BMI PGS	-0.027	0.008	-3.415	**0.0036***	-0.032	0.008	-4.255	**<0.001***
	Low AN PGS and low BMI PGS - Low AN PGS and high BMI PGS	-0.033	0.004	-9.057	**<0.001***	-0.027	0.003	-7.892	**<0.001***
	High AN PGS and low BMI PGS - High AN PGS and high BMI PGS	-0.033	0.008	-3.913	**<0.001***	-0.032	0.008	-4.043	**<0.001***
	High AN PGS and low BMI PGS - Low AN PGS and high BMI PGS	-0.038	0.004	-8.514	**<0.001***	-0.027	0.004	-6.310	**<0.001***
	High AN PGS and high BMI PGS - Low AN PGS and high BMI PGS	-0.005	0.008	-0.631	0.92	0.005	0.008	0.602	0.93
**BMD**	Low AN PGS and low BMI PGS - High AN PGS and low BMI PGS	0.007	0.003	2.037	0.17	-0.003	0.003	-0.771	0.87
	Low AN PGS and low BMI PGS - High AN PGS and high BMI PGS	-0.021	0.008	-2.687	**0.04***	-0.018	0.007	-2.492	0.06
	Low AN PGS and low BMI PGS - Low AN PGS and high BMI PGS	-0.029	0.003	-8.308	**<0.001***	-0.023	0.003	-6.790	**<0.001***
	High AN PGS and low BMI PGS - High AN PGS and high BMI PGS	-0.027	0.008	-3.399	**0.0038***	-0.016	0.008	-2.032	0.17
	High AN PGS and low BMI PGS - Low AN PGS and high BMI PGS	-0.036	0.004	-8.243	**<0.001***	-0.021	0.004	-4.820	**<0.001***

	High AN PGS and high BMI PGS - Low AN PGS and high BMI PGS	-0.008	0.008	-0.997	0.75	-0.005	0.008	-0.603	0.93
**Weight**	Low AN PGS and low BMI PGS - High AN PGS and low BMI PGS	0.008	0.008	0.923	0.79	0.008	0.008	0.923	0.79
	Low AN PGS and low BMI PGS - High AN PGS and high BMI PGS	-0.096	0.019	-4.996	**<0.001***	-0.096	0.019	-4.996	**<0.001***
	Low AN PGS and low BMI PGS - Low AN PGS and high BMI PGS	-0.107	0.009	-12.340	**<0.001***	-0.107	0.009	-12.340	**<0.001***
	High AN PGS and low BMI PGS - High AN PGS and high BMI PGS	-0.104	0.020	-5.134	**<0.001***	-0.104	0.020	-5.134	**<0.001***
	High AN PGS and low BMI PGS - Low AN PGS and high BMI PGS	-0.115	0.011	-10.605	**<0.001***	-0.115	0.011	-10.605	**<0.001***
	High AN PGS and high BMI PGS - Low AN PGS and high BMI PGS	-0.011	0.020	-0.536	0.95	-0.011	0.020	-0.536	0.95
**Height**	Low AN PGS and low BMI PGS - High AN PGS and low BMI PGS	0.001	0.002	0.415	0.98	-0.001	0.002	-0.586	0.94
	Low AN PGS and low BMI PGS - High AN PGS and high BMI PGS	0.002	0.004	0.560	0.94	0.002	0.005	0.519	0.95
	Low AN PGS and low BMI PGS - Low AN PGS and high BMI PGS	-0.002	0.002	-0.754	0.87	-0.006	0.002	-2.819	**0.03***
	High AN PGS and low BMI PGS - High AN PGS and high BMI PGS	0.002	0.005	0.360	0.98	0.004	0.005	0.748	0.88
	High AN PGS and low BMI PGS - Low AN PGS and high BMI PGS	-0.002	0.002	-0.928	0.79	-0.005	0.003	-1.784	0.28
	High AN PGS and high BMI PGS - Low AN PGS and high BMI PGS	-0.004	0.005	-0.851	0.83	-0.009	0.005	-1.717	0.32

AN = anorexia nervosa; PGS = polygenic score; BMI = body mass index (weight in kilograms/height^2 in meters); FMI = fat mass index (fat mass in kilogram /height^2 in meters); LMI = lean mass index (lean mass in kilograms/height^2 in meters); BMD = bone mineral density (gram/cm^2).

[a] Full description of the Avon Longitudinal Study of Parents and Children is described elsewhere; [2–6].

[b] Categorical variable derived from dichotomizing the AN-PGS and the BMI-PGS. For both the AN and the BMI PGS, individuals with PGS scores at or greater than the 8th decile point were regarded as the "high PGS group" while those with scores lower were considered the "low PGS group". From the dichotomized AN-PGS and BMI-PGS we were able to create a categorical variable with two levels (1) high AN-PGS and high BMI-PGS (2) high AN-PGS and low BMI-PGS.

[c] Post-hoc comparisons corrected for multiple testing using Tukey's adjustment.

* Significant after accounting for multiple testing.

Supplementary material and methods

Genotyping quality control checks

Individuals with disproportionate levels of individual missingness (i.e., >3%), insufficient sample replication (identity by descent < 0.1), biological sex mismatch, and non-European ancestry (as defined by multi-dimensional scaling using the HapMap Phase II, release 22, reference populations) were excluded. Single nucleotide polymorphisms (SNPs) with a minor allele frequency (MAF) of < 1%, excessive missingness (i.e., call rate < 95%), or a departure from the Hardy–Weinberg equilibrium (P value $< 5 \times 10^{-7}$) were removed. Imputation was conducted with Impute3 using the HRC 1.0 as the reference panel [1] and phasing was carried out using ShapeIT (v2.r644). Finally, post-imputation quality control checks were performed; any SNPs with MAF less than 1%, Impute3 information quality metric of < 0.8, and not confirming to Hardy-Weinberg equilibrium ($P < 5 \times 10^{-7}$) were removed. After data cleaning, a total of 8,654 individuals (4,225 females and 4,429 males) and 4,054,653 SNPs remained eligible for analyses.

Post-hoc power analysis

Power analysis were conducted using the polygenescore function in the AVENGEME package in R [8]. The following parameter were fixed for the power calculation of the anorexia nervosa PGS; number of independent SNPs = 46399; sample size training sample (sample size of the anorexia nervosa GWAS) = 72517 [9]; Proportion of variance explained by genetic effects in the training sample = 0.14 [9]; Proportion of markers with no effect on the training trait = 0.95; Estimated effect sizes are used as weights = TRUE; Training trait is binary = TRUE; Sampling = 0.31[9]; Effect sizes are to be shrunk to BLUPs = TRUE. The covariance between genetic effect sizes in the two samples (between anorexia nervosa and the trajectory

traits) were based on published genetic correlations for AN with BMI, FMI, LMI, BMD, weight, and height in the AN GWAS [9].

For the power calculation of the BMI PGS the following parameters were fixed: number of independent SNPs = 46399; sample size training sample = 700000; Proportion of variance explained by genetic effects in the training sample = 0.22; Proportion of markers with no effect on the training trait = 0.95; Estimated effect sizes are used as weights = TRUE; Training trait is binary = FALSE; Effect sizes are to be shrunk to BLUPs = TRUE. The covariance between genetic effect sizes in the two samples (between BMI and the trajectory traits) were either based on published genetic correlations for BMI with BMI[10], FMI, LMI, BMD [11], weight, and height or based on available online genetic correlations at https://ukbb-rg.hail.is/.

Supplemental references

1. McCarthy, S., Das, S., Kretzschmar, W., Delaneau, O., Wood, A.R., Teumer, A., Kang, H.M., Fuchsberger, C., Danecek, P., Sharp, K., et al. (2016). A reference panel of 64,976 haplotypes for genotype imputation. Nat. Genet. *48*, 1279–1283.

2. Fraser, A., Macdonald-wallis, C., Tilling, K., Boyd, A., Golding, J., Davey smith, G., Henderson, J., Macleod, J., Molloy, L., Ness, A., et al. (2013). Cohort profile: The avon longitudinal study of parents and children: ALSPAC mothers cohort. Int. J. Epidemiol. *42*, 97–110.

3. Boyd, A., Golding, J., Macleod, J., Lawlor, D.A., Fraser, A., Henderson, J., Molloy, L., Ness, A., Ring, S., and Smith, G.D. (2013). Cohort profile: The 'Children of the 90s'-The index offspring of the avon longitudinal study of parents and children. Int. J. Epidemiol. *42*, 111–127.

4. Golding, Pembrey, Jones, and The Alspac Study Team (2001). ALSPAC-The Avon Longitudinal Study of Parents and Children. Paediatr. Perinat. Epidemiol. *15*, 74–87.

5. Golding, J. (2004). The Avon Longitudinal Study of Parents and Children (ALSPAC)--study design and collaborative opportunities. Eur. J. Endocrinol. U119–U123.

6. Northstone, K., Lewcock, M., Groom, A., Boyd, A., Macleod, J., Timpson, N., and Wells, N. (2019). The Avon Longitudinal Study of Parents and Children (ALSPAC): an update on the enrolled sample of index children in 2019. Wellcome Open Res. *4*, 51.

7. Herle, M., Micali, N., Abdulkadir, M., Loos, R., Bryant-Waugh, R., Hübel, C., Bulik, C.M., and De Stavola, B.L. (2020). Identifying typical trajectories in longitudinal data: modelling strategies and interpretations. Eur. J. Epidemiol. *35*, 205–222.

8. Dudbridge, F. (2013). Power and Predictive Accuracy of Polygenic Risk Scores. PLoS Genet. *9*, e1003348.

9. Watson, H.J., Yilmaz, Z., Thornton, L.M., Hübel, C., Coleman, J.R.I.I., Gaspar, H.A., Bryois, J., Hinney, A., Leppä, V.M., Mattheisen, M., et al. (2019). Genome-wide association study identifies eight risk loci and implicates metabo-psychiatric origins for anorexia nervosa. Nat. Genet. *51*, 1207–1214.

10. Yengo, L., Sidorenko, J., Kemper, K.E., Zheng, Z., Wood, A.R., Weedon, M.N., Frayling, T.M., Hirschhorn, J., Yang, J., and Visscher, P.M. (2018). Meta-analysis of genome-wide association studies for height and body mass index in ∼700000 individuals of European ancestry. Hum. Mol. Genet. *27*, 3641–3649.

11. Liang, X., Wu, C., Zhao, H., Liu, L., Du, Y., Li, P., Wen, Y., Zhao, Y., Ding, M., Cheng, B., et al. (2018). Assessing the genetic correlations between early growth parameters and bone mineral density: A polygenic risk score analysis. Bone *116*, 301–306.
